# Developmental Dynamics of Long Noncoding RNA Expression during Sexual Fruiting Body Formation in Fusarium graminearum

**DOI:** 10.1128/mBio.01292-18

**Published:** 2018-08-14

**Authors:** Wonyong Kim, Cristina Miguel-Rojas, Jie Wang, Jeffrey P. Townsend, Frances Trail

**Affiliations:** a Department of Plant Biology, Michigan State University, East Lansing, Michigan, USA; b Department of Biostatistics, Yale University, New Haven, Connecticut, USA; c Department of Ecology and Evolutionary Biology, Yale University, New Haven, Connecticut, USA; d Department of Plant, Soil and Microbial Sciences, Michigan State University, East Lansing, Michigan, USA; Universidad de Córdoba

**Keywords:** Fusarium graminearum, Xrn1 exonuclease, fruiting body, long noncoding RNAs, perithecia, sexual development

## Abstract

Long noncoding RNA (lncRNA) plays important roles in sexual development in eukaryotes. In filamentous fungi, however, little is known about the expression and roles of lncRNAs during fruiting body formation. By profiling developmental transcriptomes during the life cycle of the plant-pathogenic fungus Fusarium graminearum, we identified 547 lncRNAs whose expression was highly dynamic, with about 40% peaking at the meiotic stage. Many lncRNAs were found to be antisense to mRNAs, forming 300 sense-antisense pairs. Although small RNAs were produced from these overlapping loci, antisense lncRNAs appeared not to be involved in gene silencing pathways. Genome-wide analysis of small RNA clusters identified many silenced loci at the meiotic stage. However, we found transcriptionally active small RNA clusters, many of which were associated with lncRNAs. Also, we observed that many antisense lncRNAs and their respective sense transcripts were induced in parallel as the fruiting bodies matured. The nonsense-mediated decay (NMD) pathway is known to determine the fates of lncRNAs as well as mRNAs. Thus, we analyzed mutants defective in NMD and identified a subset of lncRNAs that were induced during sexual development but suppressed by NMD during vegetative growth. These results highlight the developmental stage-specific nature and functional potential of lncRNA expression in shaping the fungal fruiting bodies and provide fundamental resources for studying sexual stage-induced lncRNAs.

## IMPORTANCE

Fusarium graminearum is the causal agent of the head blight on our major staple crops, wheat and corn. The fruiting body formation on the host plants is indispensable for the disease cycle and epidemics. Long noncoding RNA (lncRNA) molecules are emerging as key regulatory components for sexual development in animals and plants. To date, however, there is a paucity of information on the roles of lncRNAs in fungal fruiting body formation. Here we characterized hundreds of lncRNAs that exhibited developmental stage-specific expression patterns during fruiting body formation. Also, we discovered that many lncRNAs were induced in parallel with their overlapping transcripts on the opposite DNA strand during sexual development. Finally, we found a subset of lncRNAs that were regulated by an RNA surveillance system during vegetative growth. This research provides fundamental genomic resources that will spur further investigations on lncRNAs that may play important roles in shaping fungal fruiting bodies.

## INTRODUCTION

Genomes of eukaryotes—from simple yeast to animals—are pervasively transcribed from noncoding intergenic regions and in antisense orientation from genic regions (1, 2). Long noncoding RNAs (lncRNAs) are loosely defined as noncoding transcripts longer than 200 nucleotides, which are mostly transcribed by RNA polymerase II and share common features with mRNAs other than protein-coding capacity (3). lncRNAs are versatile molecules that not only regulate gene expression, but also affect enzymatic activities and chromosome conformation (4, 5). Since the discovery of the *XIST* lncRNA required for X chromosome inactivation (6), the roles of lncRNAs in developmental processes such as embryogenesis and tissue differentiation have been extensively studied in animals along with the advent of transcriptome sequencing (RNA-seq) technologies (7, 8). Yet the full scope of the developmental roles of lncRNAs is far from understood.

In the highly divergent yeasts Saccharomyces cerevisiae (budding yeast) and Schizosaccharomyces pombe (fission yeast), the onset of sexual sporulation and the following meiotic divisions are tightly regulated by elaborate mechanisms involving lncRNAs (9). In budding yeast, a promoter-derived lncRNA suppresses the expression of *IME1* (inducer of meiosis 1), the master regulator for the sexual sporulation, by inducing heterochromatin formation in the promoter region of *IME1* during vegetative growth (10). In addition, the transcription of another lncRNA antisense to *IME4* gene inhibits the expression of *IME4* by antagonizing sense transcription (11, 12). Although there is no such conserved or analogous regulatory mechanism in fission yeast, lncRNAs also play diverse roles in sexual sporulation: for example, sequestering RNA elimination factors that repress meiotic gene expression (13–15) and contributing to homologous chromosome pairing (16). Despite the growing evidence of the regulatory roles in yeasts, information on lncRNA expression and function during fruiting body formation in filamentous fungi is scarce.

RNA quality control mechanisms are crucial for the regulation of lncRNA expression in budding yeast. The nuclear exosome is engaged in RNA processing and degradation of transcripts, including lncRNAs that are specifically expressed during sexual sporulation; the deletion of *RRP6* encoding the exosome-associated exonuclease resulted in the accumulation of noncoding transcripts that otherwise remained silenced during vegetative growth (17–19). In human cells, promoter-derived transcripts were also ectopically expressed upon deletion of the exosome components, including the homologous *RRP6* gene, suggesting the conserved role of the exosome for lncRNA expression in diverse eukaryotes (20). The nonsense-mediated decay (NMD) pathway is another quality control checkpoint for aberrant transcripts in the cytoplasm and recently emerged as a key player for fine-tuning of both coding and noncoding gene expression (21). A genome-wide survey of human lncRNA sequences showed that most lncRNAs harbor short open reading frames (ORFs) that would lead to activation of the NMD pathway (22). In fact, subsets of lncRNAs found in budding yeast, the model plant *Arabidopsis*, and animals are subject to degradation through the NMD pathway (23–25).

The cytoplasmic exonuclease Xrn1 is the final enzyme responsible for the degradation of decapped and deadenylated transcripts that have been recognized and processed by NMD components. The deletion of *XRN1* in budding yeast also leads to the accumulation of more than a thousand cryptic noncoding transcripts termed “XUTs” (Xrn1-sensitive unstable transcripts), most of which are distinct from the noncoding transcripts that arise by exosome depletion (26). Many XUTs are antisense to annotated genes and seemed to have repressive roles in sense transcription by modulating chromatin status of the promoter regions (26).

It has been argued that organismal complexity is correlated with expression dynamics of noncoding transcripts (5, 27–29). In the kingdom Fungi, multicellular fruiting bodies have independently evolved at least twice in the diverging lineages (30, 31). Given the key regulatory roles of lncRNAs in sexual sporulation and morphological transition in yeasts (32, 33), lncRNAs may have exerted their roles in evolution of multicellularity and sexual development in filamentous fungi. Fusarium graminearum is a plant-pathogenic fungus infecting our staple crops, such as wheat and corn, and thus has been a model for studying the developmental process of perithecia, the sexual fruiting bodies of the fungus, as well as other interesting aspects of biology, including host-pathogen interaction and mycotoxin production (34, 35). The fungus is probably the best organism for investigating the lncRNA catalog in fruiting body-forming fungi, as perithecia develop at sufficient synchronicity in culture media, enabling time-series transcriptome analyses with this microscopic organism (36–38). Also, the genome sequence assembly is complete, featuring a total of 4 chromosomes (39), and the genome has been annotated and curated—although it still lacks lncRNA annotations (40, 41). In addition, plentiful genetic resources have accumulated through large-scale functional studies of perithecium development (38, 42–47).

The goal of the present study was to characterize lncRNAs that are specifically expressed in the fungal fruiting body undergoing sexual development and to investigate their developmental stage-specific expression and regulation. We identified lncRNAs with a pipeline that constructs *de novo* transcript annotations by combining RNA-seq data from vegetative and sexual developmental stages and then removes those with detectable protein-coding potential or a monotonous expression profile. Hundreds of lncRNAs that exhibit dynamic expression patterns were found, thereby expanding the universe of genomes known to have significant noncoding roles in development—specifically, here, the multicellular development of fungal fruiting bodies.

## RESULTS

### Transcriptional reprogramming during perithecium formation.

**FIG 1:**
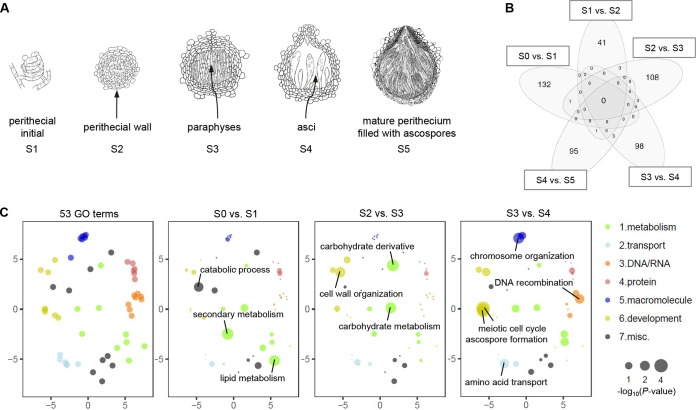
Transcriptome of F. graminearum perithecia. (A) Emergence of new tissues at the defined developmental stages during perithecial formation (S1 to S5 [not drawn to scale]). (B) Venn diagram showing the number of differentially expressed (DE) genes between two successive developmental stages (>4-fold; 5% FDR). Note that most of the DE genes were unique in each comparison. (C) Functional enrichment analyses for DE genes between two successive developmental stages. Fifty-three GO terms—which can be broadly categorized into 7 biological processes—were assessed for degree of functional enrichment and were projected to two-dimensional semantic spaces. Only GO terms with a *P* value of <0.05 are depicted in each panel.

To obtain time course transcriptome data during the sexual development of F. graminearum, we sequenced samples from hyphae, strands of cells that make up vegetative stages of most of the fungi (S0), and from five successive sexual stages (S1 to S5 [Fig. 1A]) that capture key morphological transitions during the development of perithecia (48), defined at the formation of the following: S1, perithecial initials (hyphal curls that give rise to the perithecial tissues); S2, perithecial walls; S3, paraphyses (sterile cells supporting perithecia); S4, asci (saclike structures in which ascospores develop); and S5, ascospores (meiospores). A total of 480 million RNA-seq reads were generated from 18 samples (6 stages × 3 replicates), and there were an average of 25 million mapped reads per sample (see Fig. A in Text S1 in the supplemental material). We validated our sampling scheme by perithecial morphology for 3 biological replicates, using the BLIND program (49), which determined the sequence of the developmental time course data without prior information other than gene expression data (see Fig. B in Text S1).

10.1128/mBio.01292-18.1 TEXT S1  Supplemental methods, figures, and tables. Download TEXT S1, PDF file, 2.6 MB. Copyright © 2018 Kim et al. 2018 Kim et al. This content is distributed under the terms of the Creative Commons Attribution 4.0 International license.

Differentially expressed (DE) genes between any two successive developmental stages (>4-fold at a 5% false-discovery rate [FDR]) were mostly unique compared to other pairwise stage comparisons (Fig. 1B). Overrepresented Gene Ontology database (GO) terms for the stage-specific DE genes reflected key biological processes during the morphological transitions (see Table A in Text S1). For example, the GO term “lipid metabolism” had the highest representation in the “S0 versus S1” comparison, although it was statistically nonsignificant (Fig. 1C). The accumulated lipids in hyphae and perithecial initials are vital for development of paraphyses and asci (50). Perithecia dramatically increased in size and became more rigid during S2 and S3, which is accompanied by GO terms related to carbohydrate metabolism (Fig. 1C). Finally, when the asci develop from the fertile layer during S3 and S4, meiosis-related genes were significantly enriched for GO terms, including “meiotic cell cycle process” and “ascospore formation” at a 5% FDR (Fig. 1C). It is noteworthy that most of the DE genes were upregulated in the later developmental stages (see Fig. C in Text S1), indicating that gene activation is the most common means of gene regulation during sexual development.

### Identification of lncRNA in perithecia.

To discover lncRNAs expressed during perithecial development, we adopted an established protocol for novel transcript identification (51), with some modifications (see Fig. D in Text S1). First, we constructed *de novo* transcript annotations (28,872 transcripts expressed from 20,459 genomic loci) and identified potentially novel transcripts that were absent in the reference annotations (Table 1). For identification of noncoding transcripts, the coding potential of the novel transcripts was computed by using the CPAT program (52). To maximize both sensitivity and specificity for noncoding transcript detection, the program was trained on the F. graminearum genome data set, and the threshold was set to a CPAT score of 0.540 (see Fig. D in Text S1) (cf. 0.364 for humans and 0.440 for mice [53]). The transcripts with a low coding potential (CPAT score of ≤0.540) were further scanned against the Pfam and Rfam databases to filter out transcripts encoding protein domains and harboring any known structural RNA motifs, respectively (E value of <10^−10^ [Table 1]). Finally we only retained transcripts that were differentially expressed in at least one developmental stage (5% FDR), yielding a total of 547 lncRNA candidates (Table 1; see Table S1 in the supplemental material).

10.1128/mBio.01292-18.4 TABLE S1  The annotations of 547 putative lncRNAs. The data for each lncRNA are presented from left to right as follows: (i) lncRNA IDs, (ii) genomic coordinates, (iii) strandedness, (iv) transcript class codes assigned by the *gffcompare* program, (v to vii) three features used for coding probability estimation in the CPAT program, (viii) coding probability computed by the CPAT program, (ix) adjusted *P* value for differential expression analyses performed by the Ballgown program, and (x) lncRNA type (either ancRNA or lincRNA). Download TABLE S1, RTF file, 0.1 MB. Copyright © 2018 Kim et al. 2018 Kim et al. This content is distributed under the terms of the Creative Commons Attribution 4.0 International license.

**TABLE 1:** Identification of lncRNAs expressed during sexual development

Transcript class (code)^a^	No. of transcripts		
	Novel^b^	Noncoding^c^	DE^d^
Splicing variants (J)	5,185	92	17
Tandem transcripts (P)	740	388	100
Intergenic transcripts (U)	2,215	1,054	167
Antisense transcripts (X)	2,892	1,040	263
Sum	11,032	2,574	547

a Transcript class codes were tagged by the *gffcompare* program (122).

b Among a total of 11 transcript class codes, transcripts tagged with the class codes J, P, U, and X were considered novel transcripts (see Text S1 for details).

c Noncoding transcripts were identified by the coding potential assessment tool (CPAT) program (52) and further filtered by Pfam and Rfam database searches.

d Differentially expressed (DE) noncoding transcripts of at least one developmental stage were identified as F. graminearum lncRNAs.

**FIG 2:**
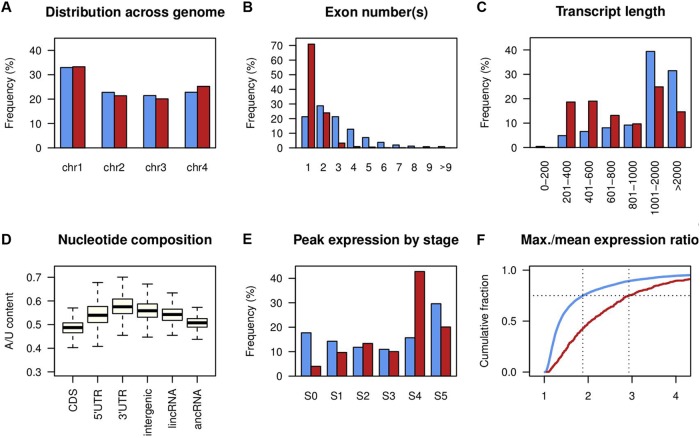
Genomic features of F. graminearum lncRNA. (A) Distribution of mRNA (blue bars) and lncRNA (red bars) across the four chromosomes. (B) Distribution of exon numbers per transcript. (C) Transcript length distribution. (D) A/U content of mRNA coding regions (CDS), 5′ untranslated regions (UTRs), 3' UTRs, intergenic regions, long intergenic lncRNA (lincRNA), and antisense lncRNA (ancRNA). Box and whisker plots indicate the median, interquartile range between the 25th and 75th percentiles (box), and 1.5 interquartile range (whisker). (E) Distribution of developmental stages at which mRNA and lncRNA showed the highest expression level. (F) Cumulative distributions of ratios of maximum and mean expression values across the developmental stages. Blue lines indicate mRNA, and red lines indicate lncRNA.

The identified lncRNAs were distributed across the 4 chromosomes and were generally shorter, with fewer exons than mRNAs (transcripts with a CPAT score of >0.540) (Fig. 2A to C). Based on the relative position to mRNAs, lncRNAs can be classified as antisense lncRNA (ancRNA) or long intergenic ncRNA (lincRNA). There were 280 ancRNAs that overlapped more than 100 bp of an mRNA on the opposite strand and 237 lincRNAs that were situated between annotated genes (Table S1). The mean A/U content of ancRNA and lincRNA sequences falls between the coding sequences of the mRNAs and the intergenic regions (Fig. 2D). These distinctive genomic features of the lncRNA sequences are commonly observed in other eukaryotes (29, 54–57). However, we identified only 5 lncRNAs that showed similarity to lncRNAs in other eukaryotes (E value of <10^−10^; see Table B in Text S1), suggesting either the poor status of lncRNA annotations in filamentous fungi or a high degree of sequence divergence in fungal lncRNAs.

### Developmental expression of lncRNA.

**FIG 3:**
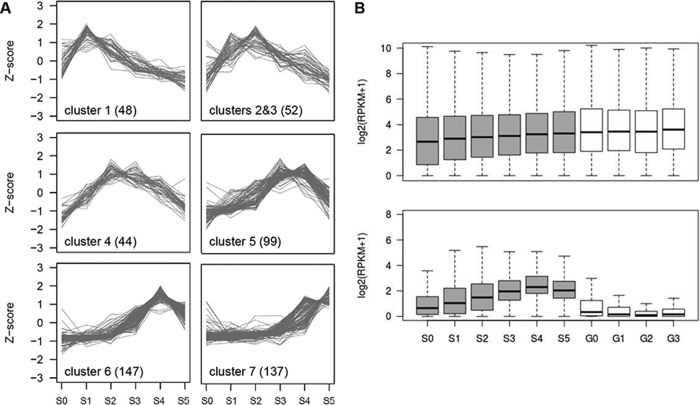
Sexual stage-induced lncRNA in F. graminearum. (A) Coexpressed clusters of lncRNAs. Trend plots of *Z*-score normalized expression values for lncRNAs (numbers in parentheses) in a given cluster are presented. (B) Expression distribution of mRNA (upper panel) and lncRNA (lower panel) for the sexual development transcriptome (gray boxes) and the vegetative growth transcriptome (white boxes). Box and whisker plots indicate the median, interquartile range between the 25th and 75th percentiles (box), and 1.5 interquartile range (whisker).

The sexual stage transcriptome data showed predominance of lncRNAs at the meiotic stage (S4), where the expression of many lncRNAs peaked (234 out of 547 [Fig. 2E]). We compared the degree of differential expression of lncRNAs to that of mRNAs (9,457 transcripts differentially expressed in at least one developmental stage at 5% FDR). The ratio of the maximum expression among six developmental stages to the mean expression over the remaining five stages was calculated for lncRNAs and the differentially expressed mRNAs. By this metric, lncRNA was prone to be more differentially expressed than mRNA (*P* = 2.2 × 10^−16^; Komolgorov-Smirnov test statistic, *D* = 0.39), with the third quartile value of the ratio measuring 2.93 for lncRNA and 1.88 for mRNA (Fig. 2F). Also, we identified seven coexpressed clusters of lncRNAs that showed developmental stage-specific expression patterns, suggesting distinct roles of lncRNAs in different stages of perithecial development (Fig. 3A).

In addition to the sexual stage data set, we obtained transcriptome data during spore germination to investigate the degree of lncRNA expression in vegetative stages. The data set was comprised of four spore germination stages: G0, fresh spore; G1, polar growth; G2, doubling of long axis; and G3, branching of hyphae (see Fig. E in Text S1). Overall expression of the lncRNA gradually increased over the course of perithecial development, peaking at S4, while most of the lncRNA remained unexpressed or had low expression in the germination stages, indicating that most of the lncRNA expression is sexual stage specific (Fig. 3B).

### Verification of lncRNA production.

**FIG 4:**
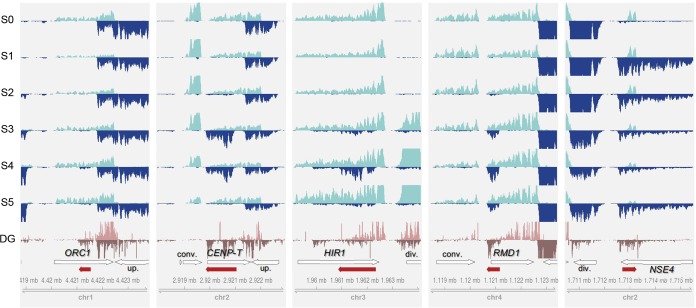
Examples of lncRNA expression across the sexual development stages. Per-base coverage of transcripts was plotted for both DNA strands in a 5-kb window. For the perithecial transcriptome data sets (S0 to S5), mapped reads of 3 biological replicates were pooled and then subsampled to 60 million reads for visual comparison of expression levels across the stages. For the degradome-seq data sets (DG), mapped reads of 2 replicates were pooled and displayed. The positions of lncRNAs (red arrows) and their neighboring genes (white arrows) are shown in the annotation track with the genome coordinate at the bottom of each panel. The genes overlapping lncRNAs on the opposite strand are labeled with abbreviated gene names in boldface: *CENP-T*, centromere protein T (FGRRES_16954), *HIR1*, histone regulatory protein 1 (FGRRES_05344); *NSE4*, nonstructural maintenance of chromosome element 4 (FGRRES_17018); *ORC1*, origin recognition complex subunit 1 (FGRRES_01336); *RMD1*, required for meiotic division 1 (FGRRES_06759). In relation to lncRNA position, neighboring genes are also labeled as follows: div., divergently transcribed gene on the opposite strand; conv., convergently transcribed gene on the opposite strand; up., upstream gene in tandem on the same strand.

**FIG 5:**
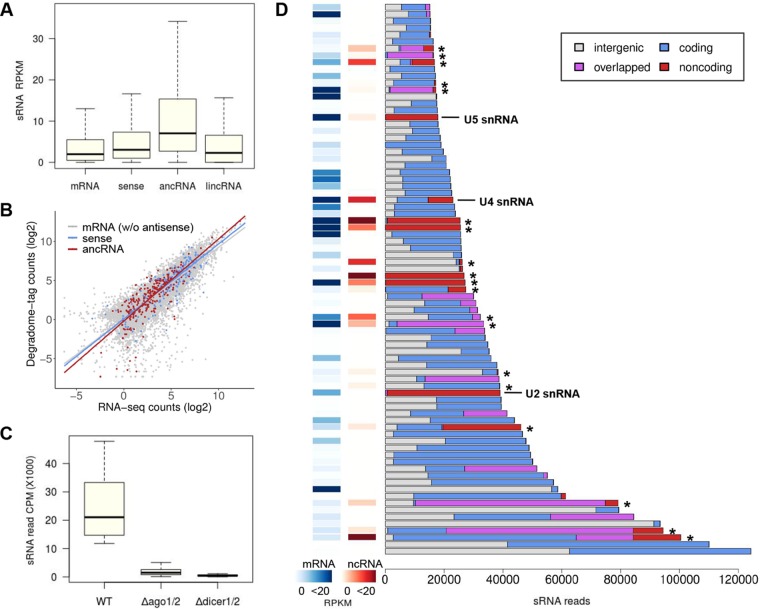
lncRNA associated with small RNA-enriched loci. (A) sRNA reads mapped to mRNAs without antisense transcripts (10,928 loci), sense mRNAs for ancRNAs (295 loci), ancRNAs (276 loci), and lincRNA (235 loci) are represented as RPKM. The number of sRNA reads aligned to ancRNA loci was more than those of the other classes of transcripts (Benjamini-Hochberg adjusted *P* value of <0.0001; Dunn’s pairwise multiple comparisons). (B) Correlation analysis between transcript abundance and degradome tag count at the meiotic stage. Lines depict regressions for different classes of transcripts. (C) sRNA reads mapped to the top 80 sRNA clusters in different genotypes are represented as counts per million (CPM). The numbers of sRNA reads mapped to the clusters were significantly reduced in the Δ*dicer1*/*2* mutant, with double deletion of Dicer genes (FGRRES_09025 and FGRRES_04408), and the Δ*ago1*/*2* mutant, with double deletion of Argonaute genes (FGRRES_16976 and FGRRES_00348) (47). (D) Fractions of sRNA reads mapped to intergenic regions (white), coding genes (blue), overlapped regions (purple), and noncoding genes (red), including lncRNAs marked with asterisks in the top 80 sRNA clusters. Overlapped regions were defined if coding or noncoding genes in the region were present on the both sides of DNA in the *de novo* annotations. Expression values (RPKM) for the closest coding gene (mRNA) to the center of each sRNA cluster are shown as heat maps, along with expression values for ncRNAs, if any, that were present in the same cluster.

To validate lncRNA expression, we chose eight lncRNAs and performed PCR with 3′ rapid amplification of cDNA ends (RACE-PCR) and Sanger sequencing. All the selected lncRNAs were amplified from total RNA extracts, and their polyadenylation sites were determined (see Fig. F in Text S1). Also, to examine if there is an intraspecific conservation in the lncRNA content and expression, we utilized degradome-seq data from another F. graminearum wild-type (WT) strain sampled at the meiotic stage (47). Degradation of lncRNA transcripts expressed at S4 was evident, which in turn confirmed the consistent lncRNA production in the two strains (Fig. 4; also see Fig. 5B below).

Fungal genomes are known for having shorter intergenic spaces than other eukaryotic genomes. Therefore, fungal lncRNAs could be a transcriptional noise arising from neighboring genes. To test this, we examined global patterns of the expression correlation between lncRNA and neighboring genes, with close examination of some selected examples whose expression was confirmed by 3′ RACE-PCR. The lncRNAs antisense to *ORC1*, *ORC2*, and *CENP-T* each had an upstream gene in close proximity on the same strand (Fig. 4; see Fig. F in Text S1). However, the sexual stage expression between the lncRNAs and their respective upstream genes was not correlated (|*r| <* 0.50 [see Table C in Text S1]). Positive correlation was observed in expression levels between lncRNAs and their divergently transcribed genes as in the *HIR1* and *NSE4* loci (*r* > 0.8 [Fig. 4; see Table C in Text S1]), indicating prevalence of bidirectional promoters for lncRNA transcription (2, 58, 59). On the other hand, the expression of lncRNAs and their convergently transcribed genes tend not to be correlated, as in the *CENP-T* and *RMD1* loci (|*r| <* 0.50 [Fig. 4; see Table C in Text S1]). These patterns were globally observed in lncRNA-associated loci (see Fig. F in Text S1), suggesting that the lncRNAs were not likely to be misannotated extensions of neighboring genes (54, 60, 61).

### Identification of sRNA-enriched loci associated with lncRNAs.

We found 300 sense mRNA-ancRNA pairs with different orientations: 5′→5′ partial, 3′→3′ partial, and full overlaps. One of the most common mechanisms involving antisense transcription is the RNA interference (RNAi) pathway incorporating small RNAs (sRNAs) generated from the double-stranded RNA regions. To investigate the degree and effect of sRNA production in the ancRNA loci, we analyzed the previously published sRNA-seq and degradome-seq data at the meiotic stage (47). As expected, sRNA reads were mapped at a higher frequency to ancRNAs than to mRNAs without overlapping antisense transcripts, the sense mRNAs, or lincRNAs (*P* < 1.2 × 10^−8^; Kruskal-Wallis test statistic, *H* = 155 [Fig. 5A]), suggesting that the ancRNA loci may serve as a major source for endogenous sRNA production. However, the correlation of the degradome-seq and our RNA-seq data at S4 showed that sRNA-mediated endonucleolytic cleavage of ancRNAs and sense mRNAs was comparable to that of mRNAs without antisense transcripts (Fig. 5B), implying that the ancRNA loci were not preferentially targeted by RNAi machinery, posttranscriptionally.

It remains paradoxical that gene silencing induced by heterochromatin formation requires sRNA production via cotranscriptional processes, sometimes from lncRNAs (62–66). To search for any lncRNAs associated with sRNA-enriched loci that could be indicative of transcriptional gene silencing events, we examined the top 80 sRNA clusters ranked by the number of mapped reads, which accounted for 62% of mapped sRNA reads (see Table D in Text S1). Production of sRNAs in the top 80 clusters was dependent on Dicers and Argonautes (47), indicating that the sRNAs were produced by RNAi machinery (Fig. 5C). Most of the sRNA clusters were found in genic regions, containing at least one annotated gene, to which a large portion of sRNA reads were mapped (Fig. 5D). We observed that the coding genes closest to the centers of sRNA clusters exhibited overall low expression (<0.5 read per kilobase per million [RPKM] in 22 out of the 80 clusters) (Fig. 5D). A significant portion of sRNAs were also derived from noncoding transcripts and genic regions overlapped with antisense transcripts, some of which were identified as lncRNAs (Fig. 5D; see Fig. S1 in the supplemental material). Unexpectedly, the lncRNAs associated with sRNA clusters exhibited moderate expression (*n =* 19; median expression of 5.2 RPKM). In addition, the coding genes closest to the centers of the sRNA clusters showed higher expression levels (*n =* 19; median expression of 4.0 RPKM) than those without an associated lncRNA (*n =* 61; median expression of 1.3 RPKM; *P* = 0.029; Mann-Whitney test statistic, *U* = 747).

10.1128/mBio.01292-18.2 FIG S1  Visualization of the expression of lncRNAs associated with sRNA clusters. Per-base coverage of transcripts was plotted for both DNA strands in an 8-kb window. For the perithecial transcriptome data sets (S0 to S5), mapped reads of 3 biological replicate samples were pooled and then subsampled to 60 million reads for visual comparison of expression levels across the stages. For the sRNA-seq data set (SR), sRNA-seq reads with T at the 5′ end and with a size ranging from 17 to 27 nt are displayed. The positions of lncRNAs (red arrows) and their neighboring genes (white arrows) are shown in the annotation track at the bottom of each panel. Download FIG S1, PDF file, 0.7 MB. Copyright © 2018 Kim et al. 2018 Kim et al. This content is distributed under the terms of the Creative Commons Attribution 4.0 International license.

### Coexpression of lncRNAs and their sense transcripts.

**FIG 6:**
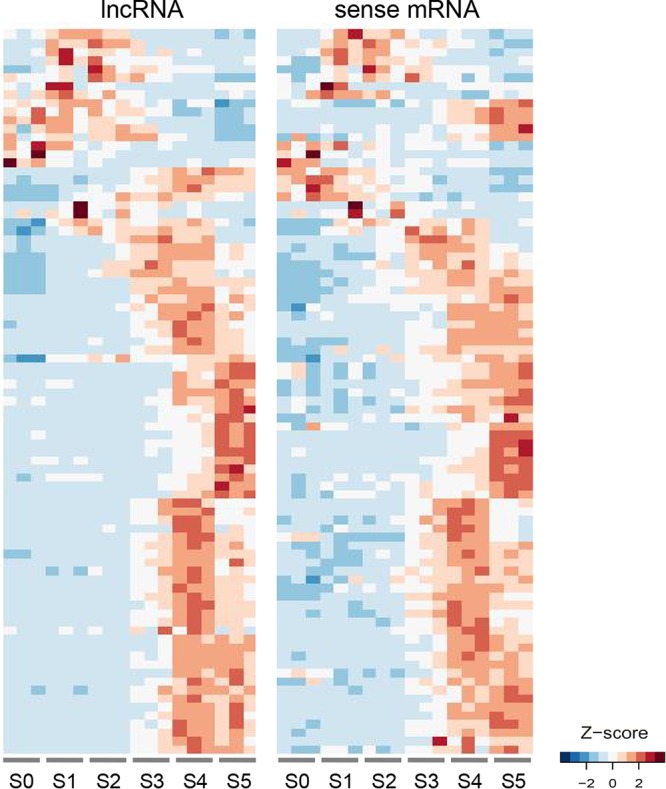
Parallel induction of sense mRNA and antisense lncRNA pairs during sexual development. Expression data of sense mRNA and antisense lncRNA pairs in 18 samples for the perithecia transcriptome (S0 to S5) were reordered by the BLIND program. The RPKM values of sense mRNA and antisense lncRNA pairs with absolute Pearson’s correlation greater than 0.7 (*P* < 0.05; Fisher’s exact test) were clustered by Euclidean distance, and heat maps of *Z*-score normalized RPKM values are presented.

We could not find strong evidence for sRNA-mediated transcriptional and posttranscriptional gene silencing in the ancRNA loci. Interestingly, we did observe gene expression correlation in many ancRNAs and sense mRNA pairs across the sexual stages (85 out of the 300 pairs with Pearson’s correlation |*r*| > 0.70; Fisher’s exact test, *P* < 0.05 [Fig. 6]), most of which were positively correlated (76 out of the 85 pairs). We asked whether the ancRNAs are antisense to genes involved in a specific biological process. We found that 17 genes in the ancRNA loci were involved in DNA metabolic processes such as DNA replication, repair, and recombination (Table 2). Notably, the positively correlated sense mRNAs were enriched for the GO term “DNA metabolism” at a 5% FDR (8 out of the 76 genes [see Table E in Text S1]), and among them, five genes (FGRRES_00736, FGRRES_01614, FGRRES_06286, FGRRES_07280, and FGRRES_08658) appeared to have functions related to DNA damage repair (Table 2). In addition, we surveyed previously published research for the 295 sense genes to determine if there are any genes known to affect sexual development. We found one gene, *RGSC* (FGRRES_09585; encoding a regulator of G proteins), whose knockout phenotype was defective in ascospore morphology (67).

**TABLE 2:** Sense transcripts encoding DNA metabolism-related gene products

Gene ID no.	Putative function	Protein domain (E value)^a^	Correlation with ancRNA^b^
FGRRES_00667	Similar to chromatin assembly complex subunit p60	Pfam12894 (3.69e−08)	**0.76** (lncRNA-026)
FGRRES_00736	Similar to DNA damage response protein WSS1	Pfam08325 (3.75e−79)	**0.89** (lncRNA-030)
FGRRES_01336	Origin recognition complex subunit 1	Cd04720 (3.48e−37)	0.69 (lncRNA-065)
FGRRES_01614	Similar to DNA mismatch repair protein MLH3	COG0323 (5.15e−39)	**0.88** (lncRNA-083)
FGRRES_05344	Histone regulatory protein 1 (HIR1)	Pfam07569 (6.27e−99)	0.29 (lncRNA-324)
FGRRES_05737	ssDNA/RNA-binding protein	Pfam00076 (1.73e−15)	**0.91** (lncRNA-342)
FGRRES_05781	Similar to TCB2 transposase	Pfam01498 (3.32e−08)	**0.97** (lncRNA-344)
FGRRES_06122	Origin recognition complex subunit 2	Pfam04084 (3.98e−163)	0.39 (lncRNA-356)
FGRRES_06231	Origin recognition complex subunit 4	Pfam14629 (1.82e−70)	0.01 (lncRNA-367)
FGRRES_06286	Similar to DNA repair polymerase RAD30	Cd01703 (6.27e−145)	**0.75** (lncRNA-369)
FGRRES_06339	DNA primase small subunit	Pfam01896 (8.59e−42)	0.40 (lncRNA-373)
FGRRES_07280	Similar to DNA repair helicase ERCC3/RAD25	Pfam16203 (6.04e−173)	**0.70** (lncRNA-461)
FGRRES_08650	Similar to SEN1 helicase	Pfam12726 (0e+00)	0.66 (lncRNA-214)
FGRRES_08658	Similar to DNA repair endonuclease ERCC4/RAD16	Pfam02732 (2.80e−25)	**0.77** (lncRNA-216)
FGRRES_09872	Similar to DNA repair dioxygenase AlkB	COG3145 (4.37e−07)	−0.02 (lncRNA-144)
FGRRES_16954	Centromere protein T (CENP-T)	Pfam15511 (6.41e−47)	−0.36 (lncRNA-235)
FGRRES_17018	Nonstructural maintenance of chromosome element 4 (NSE4)	Pfam08743 (3.83e−45)	0.10 (lncRNA-201)

a BLAST search for associated protein domains related to DNA metabolic processes.

b Pearson’s correlation for the expression levels of sense transcripts and ancRNAs across the sexual development stages. Values greater than 0.7 are marked in boldface.

### Identification of lncRNAs affected by the NMD pathway.

**FIG 7:**
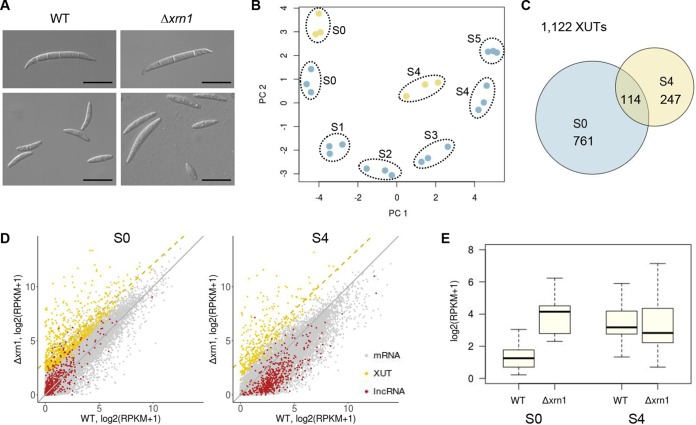
Identification of lncRNAs regulated by the NMD pathway. (A) Asexual spore (macroconidia) morphology (upper panels) and sexual spore (ascospore) morphology (lower panels). Note the size variation of ascospores in the Δ*xrn1* mutant. Scale bars = 20 µm. (B) Principal-component analysis of the perithecial transcriptome data from the wild type (S0 to S5 [blue circles]) and Δ*xrn1* transcriptome data (S0 and S4 [yellow circles]). (C) Venn diagram showing the overlap of Xrn1-sensitive unstable transcripts (XUTs) at S0 and S4 (D) Two-dimensional plots of different classes of transcripts between the wild-type (abscissa) and Δ*xrn1* mutant (ordinate) expression data at S0 and S4 (diagonal [gray line]). The yellow dashed line shows regression for the XUTs. (E) Expression distribution of 25 lncRNAs that were identified as XUTs at S0 and S4. Box and whisker plots indicate the median, interquartile range between the 25th and 75th percentiles (box), and 1.5 interquartile range (whisker).

The NMD pathway is known to regulate the expression of noncoding genes, as well as coding genes, for sexual sporulation in yeasts. To identify the sexual stage-induced lncRNAs that were affected by NMD, we generated a deletion strain lacking *XRN1* (FGRRES_06799) that encodes a 5′→3′ exonuclease. The Δ*xrn1* deletion mutant displayed a lower growth rate on different growth media compared to the wild type (WT); however, it produced normal-shaped asexual spores (Fig. 7A; see Fig. G in Text S1). We observed that perithecial development was significantly delayed and ascus development was defective in the Δ*xrn1* strain, producing variable ascospore sizes (Fig. 7A; see Fig. G in Text S1). The defective sexual phenotype was restored by genetic complementation with *XRN1* (see Fig. G in Text S1).

To identify lncRNA whose expression was affected by NMD activity, we analyzed the transcriptome data from the WT and the Δ*xrn1* strain at the vegetative (S0) and meiotic (S4) stages. The transcriptome data of the Δ*xrn1* strain were distinct from, but most similar to, those of WT at the corresponding stages, indicating drastic effects of *XRN1* on gene expression levels as a major component for RNA turnover (Fig. 7B). After expression values were normalized with 124 ribosomal protein genes that were known to be relatively insensitive to NMD activity (26), we identified a total of 1,122 XUTs that were differentially expressed at S0 or S4 in the Δ*xrn1* strain (>3-fold increase at 5% FDR [Fig. 7C; see Table S2 in the supplemental material]). After the normalization, the XUTs identified at S0 and S4 showed median 5.0- and 5.8-fold increases in the Δ*xrn1* mutant, respectively (Fig. 7D; Table S2). Many XUTs were previously annotated transcripts or isoforms of them (70% [781/1,122]), and only 11% of XUTs (122/1,122) were predicted to be noncoding transcripts (CPAT score of ≤0.540) (Table S2). Among the noncoding XUTs, we identified 25 lncRNAs whose expression was elevated upon the Xrn1 depletion at S0 and showed increasing patterns across the sexual stages (Fig. 7E; see Fig. S2 in the supplemental material). In addition, many of the coding XUTs identified at S0 were also induced as the sexual development progressed (see Fig. H in Text S1). Interestingly, we found that key components of the RNA-induced silencing complex (RISC) (68) were also identified as XUTs, such as *DICER2* (FGRRES_04408), *AGO1* (FGRRES_16976), Neurospora crassa *QIP* homologue (FGRRES_06722), and *RDRP* genes (coding for RNA-dependent RNA polymerases [FGRRES_01582, _04619, and _09076]).

10.1128/mBio.01292-18.3 FIG S2  Visualization of the expression of lncRNAs identified as XUTs. Per-base coverage of transcripts was plotted for both DNA strands in an 8-kb window. Mapped reads of 3 biological replicate samples were pooled and then subsampled to 45 million reads for visual comparison of expression levels across the stages (S0 to S5) and between the wild-type (S0) and Δ*xrn1* mutant strain (X0) at the vegetative stage. Download FIG S2, PDF file, 1.2 MB. Copyright © 2018 Kim et al. 2018 Kim et al. This content is distributed under the terms of the Creative Commons Attribution 4.0 International license.

10.1128/mBio.01292-18.5 TABLE S2  The annotations of 1,122 XUTs. The data for each XUT are presented from left to right as follows: (i) XUT IDs, (ii) genomic coordinates, (iii) strandedness, (iv) coding probability computed by the CPAT program, (v) gene IDs of reference transcripts to which XUTs overlapped more than 50% on the same strand and lncRNA IDs that were identified as XUTs, (vi) developmental stage(s) where XUTs were differentially expressed, (vii) fold change of expression levels in the Δ*xrn1* mutant at S0, (viii) adjusted *P* value for differential expression analyses at S0 performed by the Ballgown program, (ix) fold change of expression levels in the Δ*xrn1* mutant at S4, and (x) adjusted *P* value for differential expression analyses at S4 performed by the Ballgown program. Download TABLE S2, RTF file, 0.1 MB. Copyright © 2018 Kim et al. 2018 Kim et al. This content is distributed under the terms of the Creative Commons Attribution 4.0 International license.

## DISCUSSION

Here we profiled transcriptomes of vegetative and sexual stages that span the entire life cycle of F. graminearum. lncRNAs are usually an order of magnitude less abundant than mRNAs (69, 70), so the unprecedented depth of the sequencing data we generated (total of 938 million mapped reads) enabled us to capture scantly expressed noncoding transcripts. This study has revealed global properties of lncRNAs during perithecial development, characterized by dynamic and developmental stage-specific expression. In the last step of our lncRNA identification pipeline, we only included differentially expressed noncoding transcripts to discern lncRNAs of biological significance. This filtering step allowed us to identify low-abundance lncRNAs that alone could be argued to be transcriptional noise (71, 72). For many antisense lncRNAs, we detected parallel induction with their respective sense transcripts across sexual development and identified a subset of lncRNAs that are sensitive to NMD activity before sexual induction.

Our lists of F. graminearum lncRNAs contain many confident lncRNA annotations, but are still far from complete. This is primarily due to difficulty in unequivocal determination of whether a transcript is coding or noncoding (73). Although protein products from most of the bona fide lncRNAs with predicted ORFs have not been detected in cells (74, 75), many lncRNAs with one or more ORFs have been shown to be associated with ribosomes in budding yeast, *Arabidopsis*, and animals (76–81). By scrutinizing the sRNA-enriched loci, we identified several transcripts with short ORFs (ranging from 49 to 227 amino acids) as putative lncRNAs. These novel transcripts were initially filtered out by the CPAT program; however, another annotation tool, CPC2 (82), classified them as lncRNAs, and the lncRNA annotations were also supported by the lack of cross-species conservation of the deduced polypeptides (E value of ≥10^−10^ [see Table F in Text S1]).

Another source of false negatives in our data set may have arisen from poly(A)-based library preparation that excluded some lncRNAs lacking poly(A) tail. This may account for the inclusion of fewer lncRNAs in our XUT identification process, as deadenylation often takes place prior to 5′→3′ degradation by Xrn1. More precise identification of lncRNAs, especially for those undergoing deadenylation by the RNA quality control machineries, should be accompanied by cap analysis gene expression (CAGE)-seq (83–85). However, most lncRNAs identified in this study were also detected by degradome-seq (47), another sequencing method (also known as parallel analysis of RNA ends [86]), which further validates their authenticity.

To better understand how the expression of lncRNAs was controlled during perithecial development, we identified XUTs, as the NMD pathway has been shown to determine the fate of lncRNAs (21, 84). However, only 5% of the sexual stage-induced lncRNAs (25/547) were affected by Xrn1 activity, and the majority of the lncRNAs seemed to escape from the RNA surveillance system. The proportion of XUTs among the lncRNAs in F. graminearum is comparable to that in other eukaryotes, where approximately 4 to 14% of noncoding transcripts are known to undergo NMD (25). Interestingly, sexually induced RISC components were highly upregulated in the Δ*xrn1* mutant, indicating a possible link between the gene silencing pathway and the NMD pathway. Further studies on lncRNAs and the RNAi machinery that were affected by NMD activity will provide novel insights into regulatory mechanisms via altered RNA metabolism during sexual development.

We initially aimed to dissect the roles of other exosome and NMD components in lncRNA expression, such as *RRP6* (FGRRES_06049), *DBP2* (FGRRES_16145), and *MTR4* (FGRRES_01656) (84, 87). However, efforts to obtain deletion strains lacking either the *DBP2*, *MTR4*, or *RRP6* gene were unsuccessful (W. Kim and F. Trail, unpublished data), suggesting these genes may be essential in F. graminearum, as they are in another filamentous fungus, Neurospora crassa (88, 89). In fission yeast, the nuclear exosome mediates gene silencing of coding and noncoding loci during vegetative growth, and the sexual sporulation is triggered by disassembly of heterochromatin on the silenced loci (90). Therefore, it will be interesting to see if the regulation of lncRNA expression is achieved by modulation of chromatin status before and after the sexual induction in F. graminearum.

Antisense transcription can influence synthesis, expression kinetics, and stability of sense transcripts through a variety of mechanisms (5, 91). Antagonism of lncRNAs against meiotic gene expression has been documented in yeasts (10, 11, 92–95). Nevertheless, there has been growing evidence that lncRNAs activate repressed genes by modulating local chromatin structures, which can facilitate coordinated gene expression in budding yeast (96, 97) and other eukaryotes (98–101). In favor of this regulatory phenomenon, we hypothesize that the F. graminearum lncRNAs, which showed parallel induction with DNA metabolism-related genes across the sexual development stages, may play a role during meiosis that requires DNA break and repair for homologous recombination, according to “guilt by association” (1, 29). However, lncRNAs often exhibited cell-type-specific expression (100, 102, 103) and even allele-specific expression within the same nucleus (104). Therefore, we cannot rule out the possibility that the positively correlated pairs of lncRNA and sense transcript are in fact mutually exclusively expressed in different tissues or cell types in the perithecia, inhibiting each other.

Although genes found in sRNA-enriched loci are usually silenced by RNAi-dependent heterochromatin formation, some of the sRNA clusters involving lncRNAs, paradoxically, were transcriptionally active at the meiotic stage in F. graminearum. In our developmental transcriptome data, we observed that many stage-specific genes were induced over the course of sexual development, which may play key roles in the corresponding perithecial tissue development. It is conceivable that the silenced genes in vegetative tissues were derepressed by lncRNA expression by yet unknown mechanisms upon sexual induction. In N. crassa, molecular studies on the circadian clock gene locus revealed a periodic gene activation and repression mechanism involving lncRNA and facultative heterochromatin formation. The expression of an lncRNA antisense to the circadian clock gene promotes sense transcription by generating a more transcriptionally permissive chromatin that has been silenced by sRNA-mediated heterochromatin formation (105).

This study presents genome-wide characterization of lncRNAs during fruiting body development. The transcriptome landscape, including lncRNAs during the life cycle of F. graminearum, provides fundamental genomic resources to the *Fusarium* community. The detailed molecular study of newly identified lncRNAs, with its established tools for rapid genetic analyses and ample genetic resources, will contribute to the understanding of how fungi utilize noncoding genomes for laying out their multicellular body plan.

## MATERIALS AND METHODS

### Data generation and processing.

The F. graminearum genome assembly (39) and the Ensembl annotation version 32 (40) of wild-type strain PH-1 (accession no. FGSC 9075 and NRRL 31084) were used throughout this study. For total RNA extraction, synchronized fungal tissues were collected from carrot agar cultures at the previously defined developmental stages during perithecial formation (36–38, 48). For transcriptome data of spore germination stages, asexual spores (macroconidia) were spread on Bird agar medium (106) overlaid with a cellophane membrane and sampled at the indicated spore germination stages (see Fig. E in Text S1). Strand-specific cDNA libraries were constructed from poly(A)-captured RNAs, using the Kapa stranded RNA-Seq library preparation kit (Kapa Biosystems, Wilmington, MA), and sequenced on the Illumina HiSeq 2500 platform (Illumina, Inc., San Diego, CA) at the Michigan State University Research Technology Support Facility (https://rtsf.natsci.msu.edu/genomics). Following quality control for raw reads (Text S1), filtered reads were mapped to the repeat-masked genome using the HISAT2 program (v2.0.4) (107), and a genome-guided transcriptome assembly was performed using the StringTie program (v1.3.0) to generate *de novo* transcript annotations (108).

### Differential expression and functional enrichment analyses.

Read counts for gene loci were calculated using the *htseq-count* program (v0.6.1) (109). On average, 87% of mapped reads were overlapped to exons in the *de novo* annotations. Gene expression levels in counts per million (CPM) values were computed and normalized by effective library size estimated by trimmed mean of *M* values, using the edgeR R package (v3.14.0) (110). Only genes with CPM values greater than 1 in at least 3 samples were kept for further analyses (15,476 out of 20,459 gene loci). Then, differentially expressed (DE) genes showing greater than 4-fold difference at an FDR of 5% were identified between two successive developmental stages, using the limma R package (v3.28.21) (111). GO terms were assigned to the *de novo* annotations, using the Trinotate program (v3.0.1) (112). The list of GO terms was customized by adding several GO terms related to developmental processes to the GO Slim terms specific for fission yeast (113). Functional enrichment analyses for DE genes were performed using the GOseq R package (v1.24.0), including only those genes annotated by one or more GO terms (114). To assess enrichment of GO terms, the Wallenius approximation (an extension of the hypergeometric distribution) and Benjamini-Hochberg method were used to calculate the FDR-corrected *P* value.

### Conserved lncRNA search.

To search for conserved lncRNAs, the 547 lncRNAs were queried against the RNAcentral database version 5 (http://rnacentral.org), using the *nhmmer* program, which detects remote homologies, in the HMMER software (v3.1b2) (115). Search hits with an E value of <10^−10^ were reported.

### Coexpression network analysis.

The weighted gene correlation network analysis (WGCNA) R package (v1.51) (116) was used to cluster lncRNAs by averaged RPKM values for developmental stages. The “*pickSoftThreshold*” function was used to determine soft-thresholding power that measures the strength of correlation based on not just the direct correlation value of pairs of genes, but also the weighted correlations of all of their shared neighbors in the network space (117). The soft-thresholding power of 26 was selected, which is the lowest power for which the scale-free topology model fit index reaches 0.80. A range of treecut values were tested for cluster detection, and the value was set to 0.18 (corresponding to a correlation of 0.82). All other WGCNA parameters remained at their default settings.

### Identification of small RNA clusters.

The sRNA-seq and degradome-seq data were obtained from NCBI GEO (GSE87835) and NCBI SRA (PRJNA348145), respectively (47). In filamentous fungi, the size of a majority of sRNAs ranges from 17 to 27 nucleotides (nt), with a strong 5′ U preference (70 to 82%) (47, 118). Thus, clusters of 17- to 27-nt-long 5′-U sRNA reads were detected across the genome, using the ShortStack program (v3.8.2) (119) with the option arguments “--pad 22” and “--mincov 20.” Subsequently, the number of 5′ U sRNA reads aligned to different genomic features (e.g., coding regions) were counted for each sRNA cluster, using the *htseq-count* program (v0.8.0) (109). The degradome-seq data set was processed according to the previous study (47).

### Expression correlation analysis.

The expression value matrix for the 18 RNA-seq data (6 stages × 3 replicates) were rearranged by the BLIND program (see Fig. B in Text S1). Pearson’s correlation and the associated *P* value by Fisher’s exact test were calculated for the expression levels of the 300 sense mRNA-antisense lncRNA pairs, using an R script (29). For sense mRNAs only, which showed positive correlation with antisense lncRNAs, we performed functional enrichment analyses, using the GOseq R package (v1.24.0) (114), as done for DE genes between two successive developmental stages (see above).

### XUT identification.

Generation, confirmation, and complementation of the Δ*xrn1* mutant are described in Text S1, and the primers used in this study are listed in Table S3 in the supplemental material. To incorporate novel transcripts that were only expressed in the Δ*xrn1* mutant due to loss of NMD activity, the RNA-seq reads for the Δ*xrn1* mutant were independently processed, and the assembled transcripts were merged to the *de novo* annotations, using the “*merge*” function in the StringTie program (v1.3.0) (108). Transcript abundance is globally affected by *XRN1* deletion in budding yeast; however, the expression of ribosomal protein genes shows only slight increases in the Δ*xrn1* mutant and remains constant after lithium treatment, which inhibits 5′→3′ exonuclease activities (26, 120). Thus, before XUT identification, expression values were normalized in such a way that ribosomal protein genes are expressed at the same levels in the WT and Δ*xrn1* mutant by multiplying by 0.66 and 1.96 the RPKM values of the WT at S0 and S4, respectively (see Fig. I in Text S1). Differential expression analyses were performed, using the “*stattest*” function with the option argument “libadjust = FALSE” in the Ballgown R package (v2.8.4) (121). The DE transcripts showing a 3-fold increase in the Δ*xrn1* mutant were identified as XUTs (5% FDR). We excluded transcripts with relatively low expression levels in the Δ*xrn1* mutant (<3.0 RPKM) from the putative XUTs to reduce possible false positives.

10.1128/mBio.01292-18.6 TABLE S3  Primers used in this study. Download TABLE S3, DOCX file, 0.1 MB. Copyright © 2018 Kim et al. 2018 Kim et al. This content is distributed under the terms of the Creative Commons Attribution 4.0 International license.

### Accession number(s).

The RNA-seq data generated in the present work have been deposited in NCBI’s Gene Expression Omnibus (https://www.ncbi.nlm.nih.gov/geo) and are accessible through GEO series accession no. GSE109095, which is composed of the two data sets—the sexual stage and *Δxrn1* mutant data set (GSE109094) and the spore germination stage data set (GSE109088).
