# Supplementary material for: Developmental Dynamics of Long Noncoding RNA Expression during Sexual Fruiting Body Formation in Fusarium graminearum
Source: mBio. 2018 Aug 14;9(4):e01292-18. doi: 10.1128/mBio.01292-18 (PMC6094484; doi:10.1128/mBio.01292-18)
Supplement: TEXT S1 [file mbo004184025s1.pdf]

Text S1. Supplemental Methods, Figures, and Tables

Developmental dynamics of long noncoding RNA expression during sexual fruiting body formation in *Fusarium graminearum*

by

Wonyong Kim, Cristina Miguel-Rojas, Jie Wang, Jeffrey P. Townsend and Frances Trail

Table of Contents

List of Supplemental Figures 2

List of Supplemental Tables 3

Supplemental Methods. 4

Literatures Cited. 31

List of Supplemental Figures

Fig. A. RNA-seq reads mapping rates of the perithecia transcriptome dataset. 11

Fig. B. Principal components analysis using the perithecia transcriptome dataset. 12

Fig. C. MA plots for differential expression analyses. 13

Fig. D. lncRNA identification protocol 14

Fig. E. Transcriptome data for vegetative growth of *F. graminearum*. 15

Fig. F. Verification of lncRNA expression. 16

Fig. G. Generation of an *XRN1*-deletion mutant and its genetic complementation 18

Fig. H. Coexpression network analysis for XUTs. 20

Fig. I. Transcriptome data comparison for the wild-type and Δ*xrn1* mutant 21

List of Supplemental Tables

Table A. Functional enrichment analyses for differentially expressed genes

between two successive developmental stages. 22

Table B. Conserved lncRNAs in other eukaryotes. 24

Table C. Expression correlation of selected lncRNAs and neighboring genes 25

Table D. Top 80 sRNA clusters across the *F. graminearum* genome 26

Table E. Functional enrichment analyses for sense mRNAs that showed

expression correlation with antisense lncRNAs. 28

Table F. The annotations of additional lncRNAs found in sRNA clusters 30

Supplemental Methods

Fungal RNA extraction

Total RNA was extracted from hyphae and perithecia ground in liquid nitrogen using TRIzol reagent (Thermo Fisher Scientific, Waltham, MA) according to the manufacturer's instruction, with additional extraction steps: two phenol (pH 4.6)-chloroform-isoamyl alcohol (25:24:1) extraction steps followed by two chloroform extraction steps after the initial TRIzol-chloroform phase separation. RNA pellets were dissolved in 88 µL of nuclease-free water and subjected to genomic DNA digestion by DNase treatment (Qiagen, Germantown, MD). Then, RNA samples were concentrated using the RNA Clean & Concentrator (Zymo research, Irvine, CA). The quality of the RNA was confirmed using the Agilent 2100 Bioanalyzer (Agilent Technologies, Palo Alto, CA). About 2 µg of total RNA was used for cDNA library construction.

Quality control of RNA-seq data

The quality of raw reads (single-end, 50 bp) was assessed with the FastQC program (v0.11.3; www.bioinformatics.babraham.ac.uk/projects/fastqc) and poor quality reads were trimmed or filtered out, and adapters and homopolymers were trimmed from raw reads, using the ngsShoRT program (v2.2; Chen et al. 2014), with option arguments: '-lqs 12', '-tera_avg 20', '-5a_mp 98', and '-rmHP_ml 10'.

LncRNA identification procedure

To identify lncRNAs in the *de novo* annotations, we adopted an established protocol with some

modifications (Weirick et al. 2016; Fig. D). We mapped RNA-seq reads to a repeat-masked genome in the first place. By doing so, we avoid identifying transposons or repetitive DNA elements as lncRNA. Comparison of the mapping rates on the masked and unmasked genome sequences indicated that 3% of total reads were derived from repeat regions, implying that many repeat sequences are still being transcribed (Fig. A). To discern novel transcripts, the original protocol included transcripts with the transcript class codes ‘J’ and ‘U’ tagged by the *gffcompare* program (Pertea et al. 2016). Furthermore, we included transcripts with the transcript class code ‘X’ as our cDNA library preparation protocol preserved strandedness. Also, transcripts with the transcript class code ‘P’ were added to our list of novel transcripts, as these may include tandemly expressed transcripts, aside from annotated genes in the reference annotations.

The coding potential assessment tool (CPAT v1.2.2; Wang et al. 2013) was used to assess coding probability for all the transcript sequences in the *de novo* annotations, using a logistic regression model and hexamer frequency trained on *F. graminearum*. The hexamer frequency was calculated for 14,164 coding gene sequences and for 1,825 noncoding gene sequences in the reference annotations, separately. To determine the optimum cutoff value for noncoding transcript prediction, we performed 10-fold cross-validation with a set of randomly selected 1,825 coding sequences. An averaged two-graph receiver operating characteristic curve from 10 validation runs were drawn, and the coding probability threshold was determined to be 0.540 (Fig. D). To further filter out potentially coding sequences from the putative noncoding transcripts that passed the threshold (CPAT score ≤ 0.540), protein sequences of the putative noncoding transcripts were deduced, using the TransDecoder program (v3.0.0; https://transdecoder.github.

io), and were queried against the Pfam-A database (v30.0; Finn et al. 2016), using the *hmmscan* program in the HMMER software package (v3.1b2; http://hmmer.org). Also, to exclude any structural ncRNAs (e.g. tRNA, rRNA, snRNA) from our list of noncoding transcripts, sequences of the putative noncoding transcripts were queried against the Rfam-cm database (v12.1; Nawrocki et al. 2015), using the *cmscan* program in the Infernal software (v1.1.2; Nawrocki and Eddy 2013). Transcripts detected by the *hmmscan* and *cmscan* programs (E-value < 10^{-10}) were discarded from the list of noncoding transcripts.

Differentially expressed (DE) noncoding transcripts were identified, using the Ballgown R package (v2.4.2; Frazee et al. 2015). Before the analysis, transcripts with a variance of RPKM values less than one were removed, according to Pertea et al. (2016). Among the variance filtered transcripts in the *de novo* annotations, DE noncoding transcripts in at least one developmental stage were identified at 5% FDR, using the *stattest* function with option arguments: 'timecourse = TRUE' and 'df = 5', and were tentatively classified as lncRNAs.

3' rapid amplification of cDNA ends

Primers for 3' rapid amplification of cDNA ends (3' RACE) were designed (Table S3), and the experiments were performed, according to the previously published protocol with a minor modification (Scotto–Lavino et al. 2006). One microgram of the RNA samples from stages S0 and S4 were reverse-transcribed with the Qt primer (modified to improve annealing of the primer), using the SuperScript IV reverse transcriptase kit (Thermo Fisher Scientific, Waltham, MA). Subsequently, 3' ends of target genes were amplified, using Qo and gene-specific primer 1 (GSP1) pairs. Second amplifications were

performed using nested primer pairs, Qi and GSP2 to suppress the amplification of non-specific products. Amplified fragments from the second amplification (Fig. F) were then extracted and cloned into pJET1.2 vector, using the CloneJET PCR Cloning Kit (Thermo Fisher Scientific, Waltham, MA), and sequenced at Michigan State University's Research Technology Support Facility by using an ABI Prism 3730xl genetic analyzer (https://rtsf.natsci.msu.edu/genomics/sequencing-services/sanger).

Classification of lncRNAs

A genome arithmetic toolset, Bedtools, (v2.24.0; Quinlan and Hall 2010) was used to determine whether lncRNAs are ancRNAs or lincRNAs in relation to genomic coordinates of coding genes listed in the *de novo* annotation. Since a significant proportion of genes are found to be transcribed into more than one isoform in fungi (Pelechano et al. 2013), a set of the longest transcripts expressed from each gene locus were retrieved using a custom Python script. We classified 280 lncRNAs that overlapped at least 100 bp to coding transcripts (CPAT score > 0.540) on the opposite strand as ancRNAs, and classified 237 lncRNAs that did not overlap to any transcripts as lincRNA (Table S1). Thirty lncRNAs were overlapped less than 100 bp to coding transcripts or to noncoding transcripts (CPAT score ≤ 0.540), thus were not classified.

Small RNA read and degradome tag mapping

Raw data for sRNA-seq and degradome-seq data at meiotic stage were obtained from NCBI GEO (GSE87835) and NCBI SRA (PRJNA348145), respectively (Son et al. 2017). Following quality control

and adapter trimming, we aligned sRNA-seq reads and degradome-seq tags with perfect matches to the reference genome sequence, using the HISAT2 program (v.2.1.0; Kim et al. 2015), with option arguments: '--no-spliced-alignment', '--no-softclip', and '--mp 50,50'. After read mapping, we extracted the mapped reads with 17–27 nt, using the 'reformat.sh' script in BBMap tools (https://sourceforge.net/projects/bbmap), and with 'T' at the 5' end using a custom Python script. We identified genes without antisense transcripts on the opposite strand (10,928 out of 20,459 loci) in the *de novo* annotation, using the Bedtools *intersect* function (v2.24.0; Quinlan and Hall 2010), and used the *htseq-count* program (v0.8.0; Anders et al. 2015), with an option argument: '--stranded reverse' to calculate sRNA read counts for different transcript types. For degradome-seq data, mapped reads with 16 and 17 nt were extracted, according to the previous study (Son et al. 2017). To calculate degradome tag counts for mRNAs and ancRNAs, we used the *htseq-count* program (v0.8.0; Anders et al. 2015), with an option argument: '--stranded yes'.

Generation of targeted gene-deletion mutants

The double-joint PCR and split-marker strategies were employed to generate gene-deletion mutants (Catlett et al. 2003; Yu et al. 2004). Primers used in targeted gene deletion are listed in Table S3. For the first round PCR, upstream (left flanking) regions and downstream (right flanking) regions of the coding sequence of target genes were amplified, using L5 and L3 primer pairs and R5 and R3 primer pairs, respectively. L3 and R5 primers have 27 nt-long overhang sequences complementary to the 5′ and 3′ ends of a 1,376-bp hygromycin phosphotransferase gene (*hph*) cassette that was amplified from pCB1004

plasmid (Carroll et al. 1994), using HYG5 and HYG5 primers. In the second round PCR, left and right flanking regions were fused to the *hph* cassette through PCR by overlap extension (Yu et al. 2004). For the amplification of split marker constructs, the second round PCR products were used as templates for the third round PCR with nested primer pairs: N5 and HY-R pairs, and YG-F and N3 pairs.

The split marker constructs were introduced into protoplasts by polyethylene glycol-mediated genetic transformation as described in Hallen-Adams et al. (2011). Target gene replacement with the *hph* cassette was verified in transformants by PCR checks using primers outside the area of gene replacement; L5 and HY-R primer pairs (P1) for upstream regions, and YG-F and R3 primer pairs (P2) for downstream regions (Fig. G). The deletion of target genes was checked by PCRs using gene-specific primers (GSPfwd and GSPrev). Genetic complementation was accomplished by introducing the coding sequence of the *XRN1* gene including a 1.5 kb of the upstream region that had been cloned to pDS23 plasmid (Teichert et al. 2012) (Fig. G).

RNA-seq for *xrn1*-deletion mutant

To obtain transcriptome data for Δ*xrn1*, samples for hyphae stage (S0) were collected at 5 days after the growth on carrot agar (*cf*. 4 days for WT), when the fungal colony reached to the margin of plates (6 cm in diameter). Also, perithecial development in Δ*xrn1* was significantly delayed (Fig. G). Thus, samples for the meiotic stage (S4) were collected at 7 days after sexual induction (*cf*. 4 days for WT). After RNA extraction and cDNA library construction, three biological replicates for the two developmental stages were sequenced on the Illumina HiSeq 4000 platform (Illumina Inc., San Diego,

CA) at the MSU's Research Technology Support Facility. After quality control of raw reads, we obtained average 24 million mapped reads per sample.

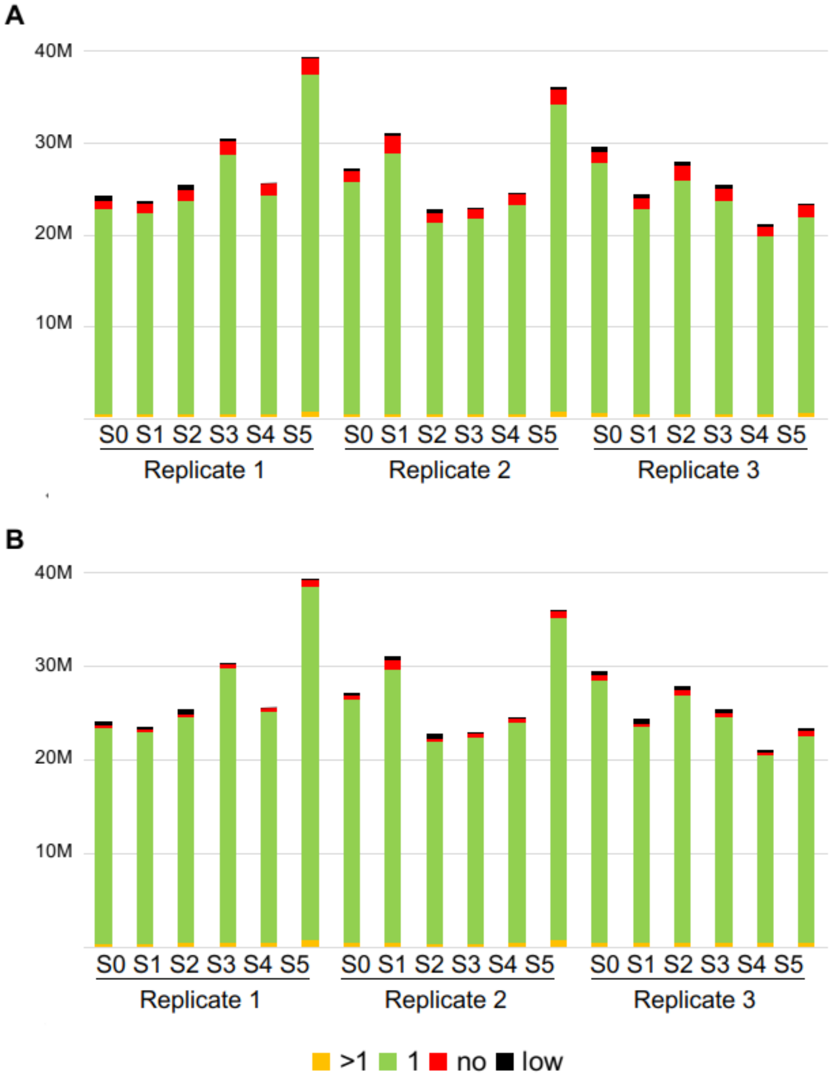

Fig. A. RNA-seq read mapping rates of the perithecia transcriptome dataset. After quality control, single-end reads were mapped to the repeat-masked genome sequence (*A*), or the unmasked genome sequence (*B*). Keys indicate as follows: >1–reads mapped on multiple loci, 1–reads mapped on single locus, no–reads not mapped, low–poor quality reads filtered before mapping.

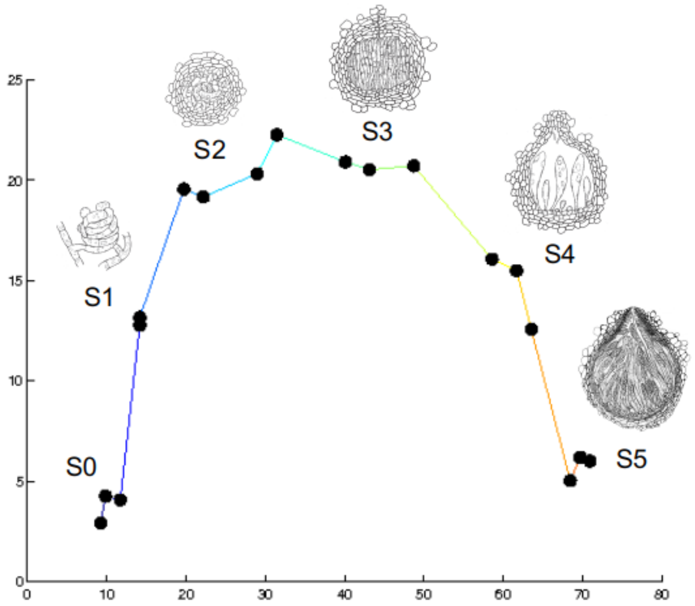

Fig. B. Principal components analysis using the perithecia transcriptome dataset. The developmental path calculated by BLIND program (Anavy et al. 2014) was connected with lines where the color indicates the relative developmental stages of the samples; the color scheme 'blue to red' corresponds to developmental stages 'earlier to later'. Developmental stages as inferred upon sample collection were drawn (not to scale) next to the corresponding sample data points. *x*-axis: the first principal component, *y*-axis: the second principal component.

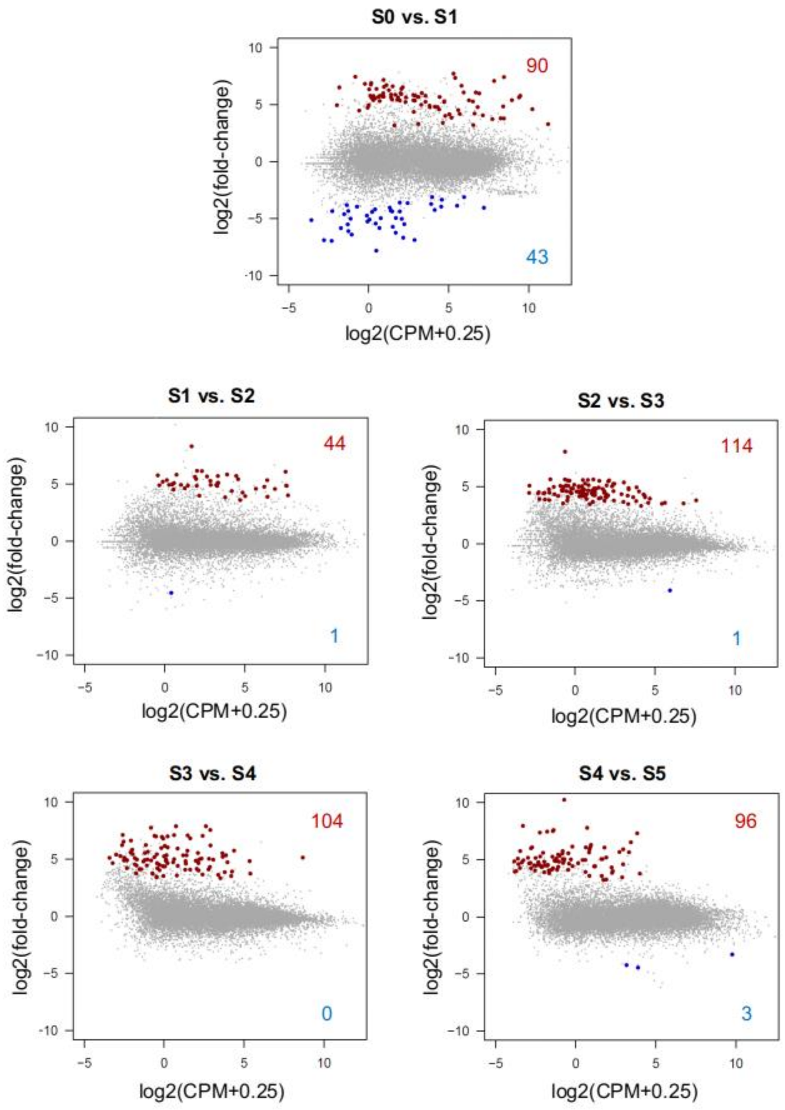

Fig. C. MA plots for differential expression analyses. Scatterplots of log2-transformed fold-change versus log2-transformed CPM were displayed for 15,476 genes in comparisons of two successive developmental stages. Up-regulated genes in the advanced stages are highlighted red, while down-regulated genes are highlighted in blue. The numbers of up- and down-regulated genes were shown in each plot.

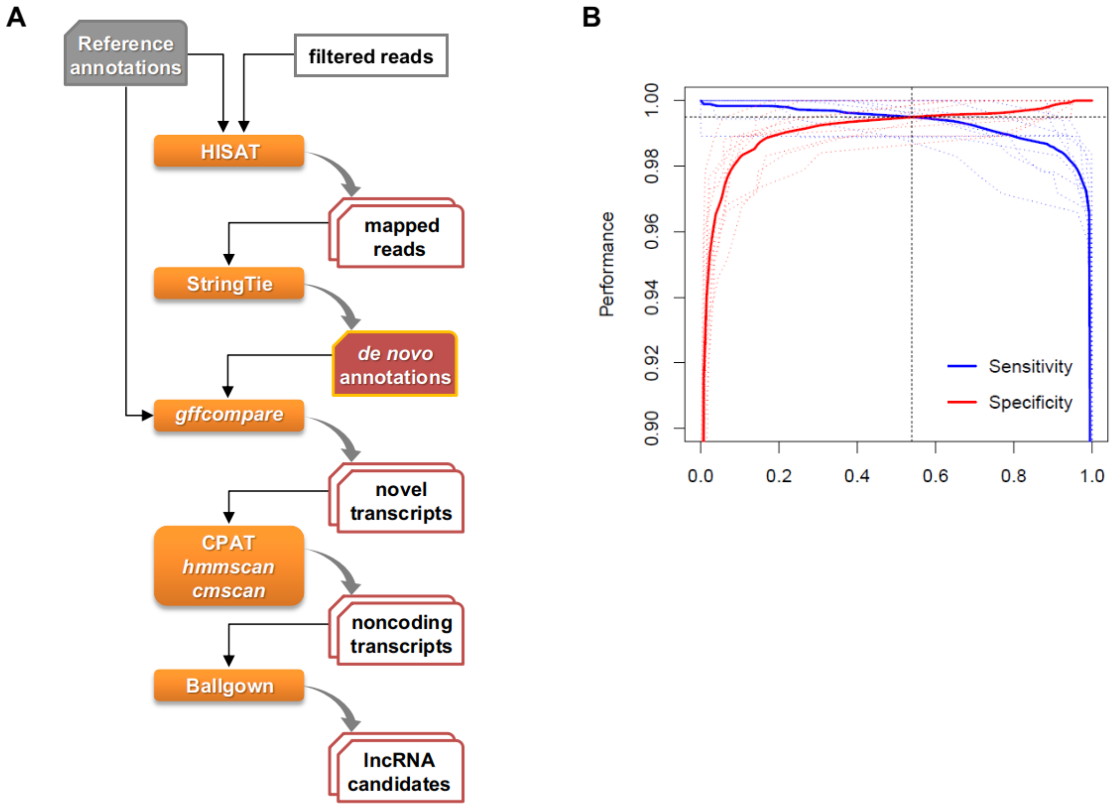

Fig. D. lncRNA identification protocol. (*A*) A bioinformatics pipeline for lncRNA discovery with RNA-seq data (see Supplemental Methods for details). (*B*) Performance evaluation of noncoding transcript prediction. Two-graph receiver operating characteristic analysis was performed to determine an optimum CPAT cutoff value for noncoding transcript calls. Dashed curves represent the 10-fold cross-validation, and solid curves represent the averaged curve from the 10 validation runs.

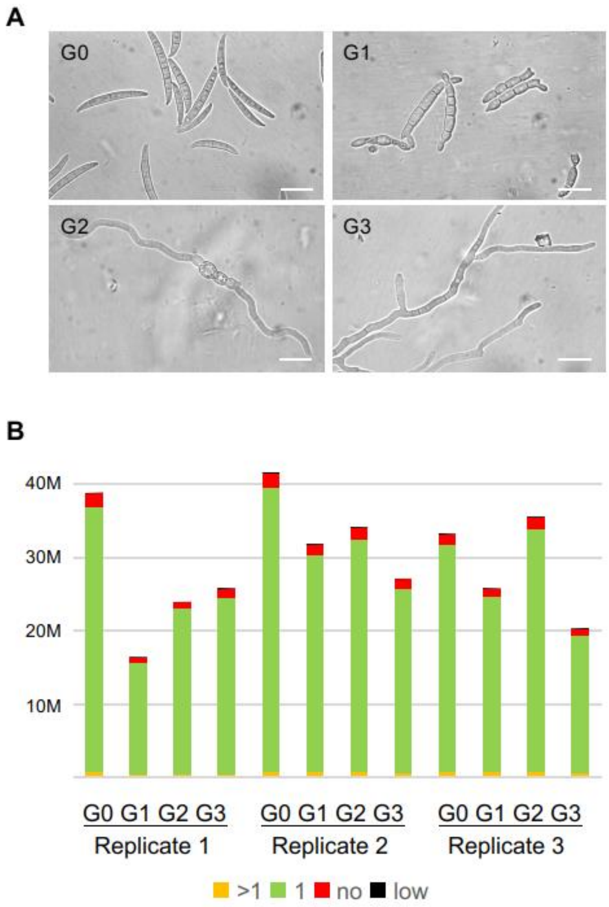

Fig. E. Transcriptome data for vegetative growth of *F. graminearum*. (*A*) Spore suspension was inoculated on Bird agar media and harvested for RNA extraction at the indicated spore germination stages: G0–fresh spore, G1–polar growth, G2–doubling of long axis, and G3–branching of hyphae. Scale bar = 20 µm. (*B*) RNA-seq reads mapping rates. After quality control, single-end reads were mapped to the repeat-masked genome sequence. Keys indicate as follows: >1–reads mapped on multiple loci, 1–reads mapped on single locus, no–reads not mapped, low–poor quality reads filtered before mapping.

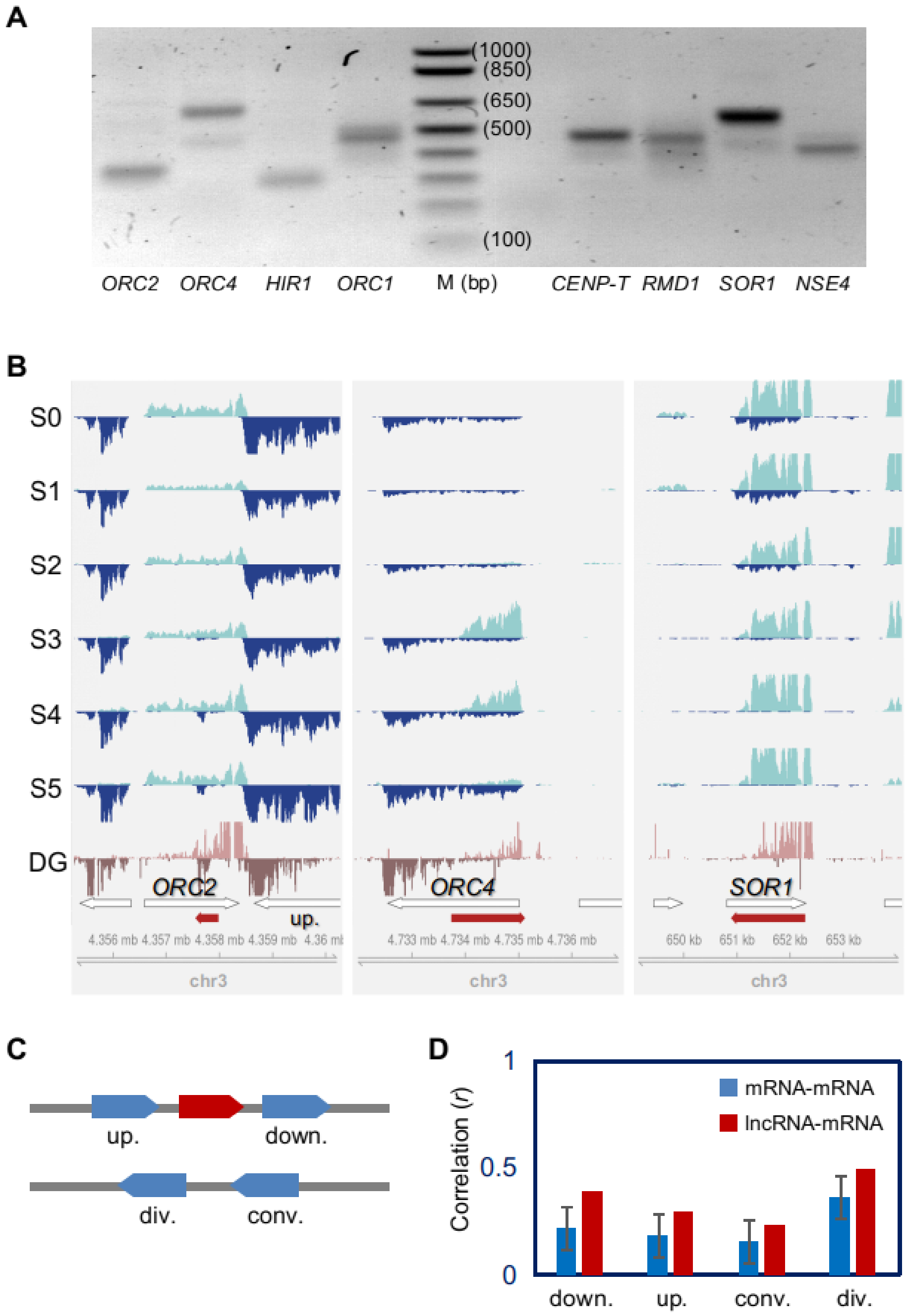

Fig. F. Verification of lncRNA expression. (*A*) 3'RACE-PCRs were performed to confirm the expression of 8 selected lncRNAs antisense to protein-coding genes, and to determine the 3' ends of the lncRNAs. Amplicons of lncRNAs are labelled as their respective sense gene names; *CENP-T*—centromere protein

T (FGRRES_16954), *HIR1*—histone regulatory protein 1 (FGRRES_05344), *NSE4*—non-structural maintenance of chromosome element 4 (FGRRES_17018), *ORC1*—origin recognition complex subunit 1 (FGRRES_01336), *ORC2*—origin recognition complex subunit 2 (FGRRES_06122), *ORC4*—origin recognition complex subunit 4 (FGRRES_06231), *RMD1*—required for meiotic division 1 (FGRRES_06759), *SOR1*—sorbitol dehydrogenase (FGRRES_04922). (*B*) RNA-seq (S0–S5) and degradome-seq (DG) reads plots for the selected lncRNAs that were not presented in Figure 4. Per-base coverage of transcripts was plotted for both DNA strands in a 5 kb window. The positions of lncRNAs (red arrows) and their neighboring genes (white arrows) are shown in the annotation track with genome coordinate at the bottom of each panel. (*C*) A schematic diagram of relative orientations for a pair of genes. div.—divergently transcribed gene on the opposite strand, conv.—convergently transcribed gene on the opposite strand, up.—upstream gene in tandem on the same strand, down.—downstream gene in tandem on the same strand. (*D*) Correlation of expression levels between neighboring genes. RPKM values across 18 samples were used to calculate the Pearson's correlation coefficient of expression levels between neighboring genes, which were re-ordered by the BLIND program (Fig. B). Shown are the average Pearson's correlation coefficients for the 547 lncRNAs and their neighboring mRNA pairs with the indicated relative orientations (red bars), considering only pairs within 1 kb of each other. For comparison, the average Pearson's correlation coefficients were calculated for five number-matched cohorts of randomly chosen mRNAs and their neighboring genes. The mean values of the results with the 5 cohorts are presented for each orientation (blue bars), with error bars showing the 95% confident interval.

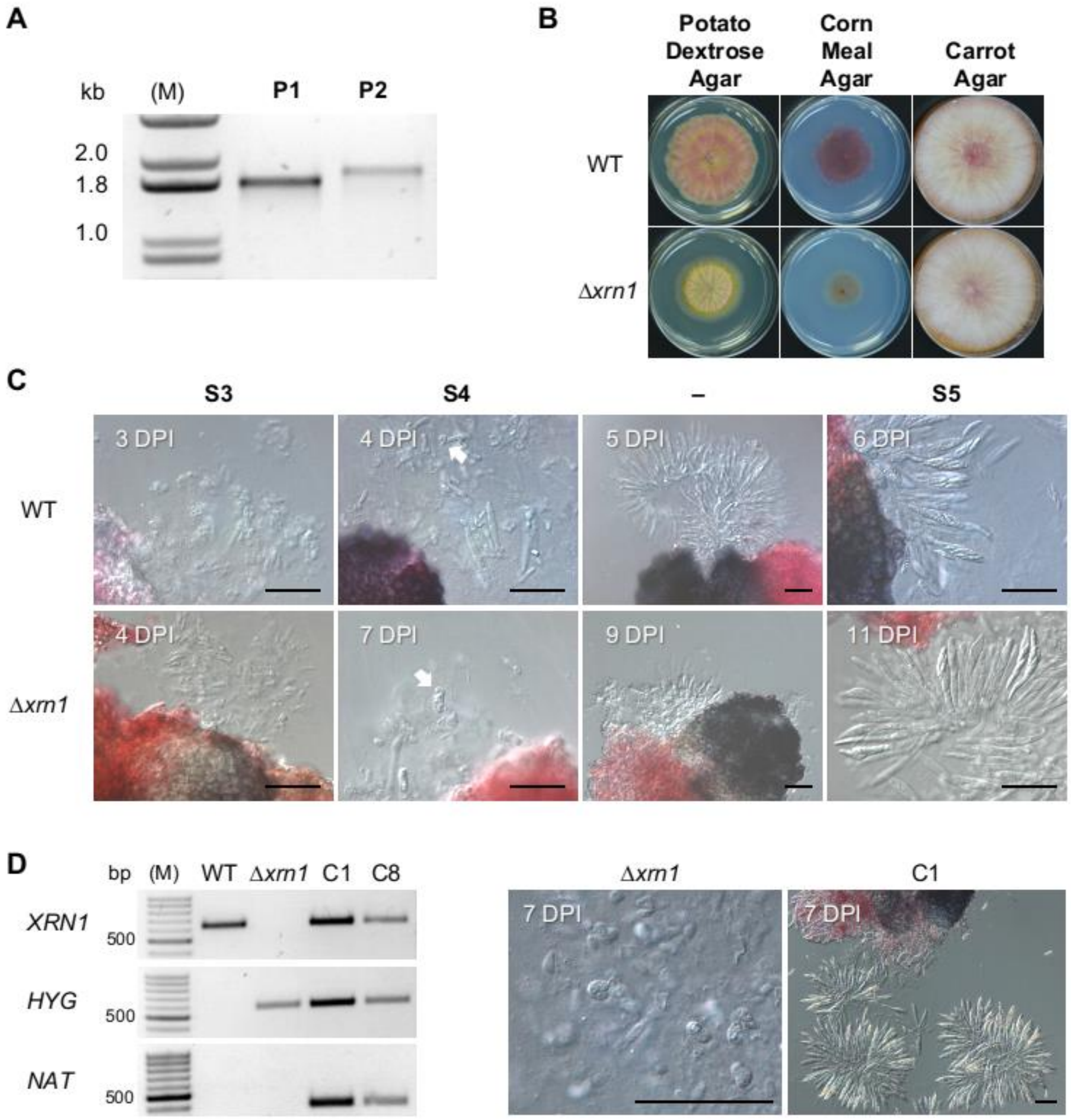

Fig. G. Generation of an *XRN1*-deletion mutant and its genetic complementation. (*A*) The PCR checks with primer pairs (P1 and P2) confirmed homologous integration of the *hph* cassette to the *XRN1* loci. M = DNA marker. (*B*) Comparison of growth rate and colony morphology between the WT and Δ*xrn1*.

Photographs were taken at 4 days after cultivation. (*C*) Perithecia squash mounts of the wild-type (WT) and Δ*xrn1* at different developmental stages (S3–S5); At S3, fragmented paraphyses cells were released from a squashed perithecia; At S4, white arrows indicate a Crozier cell from which ascus develops immediately after karyogamy; At S5, ascospores matured in asci. Note that the perithecia development was significantly delayed in Δ*xrn1*. DPI (days post sexual induction). (*D*) Genetic complementation of Δ*xrn1*. The presence of *XRN1* was confirmed by PCR with GSPfwd-xrn1 and GSPrev-xrn1 primer pairs (Table S3) in two complemented strains (C1 and C8). Also, the presence of hygromycin phosphotransferase (*HYG*) gene used as a selection marker in the gene replacement experiment and nourseothricin acetyl transferase (*NAT*) gene used as a selection maker in the gene complementation experiment were checked by PCR. Perithecia squash mounts of the Δ*xrn1* regenerated from protoplast and a complemented strain (C1) at S4. Scale bar = 50 μm.

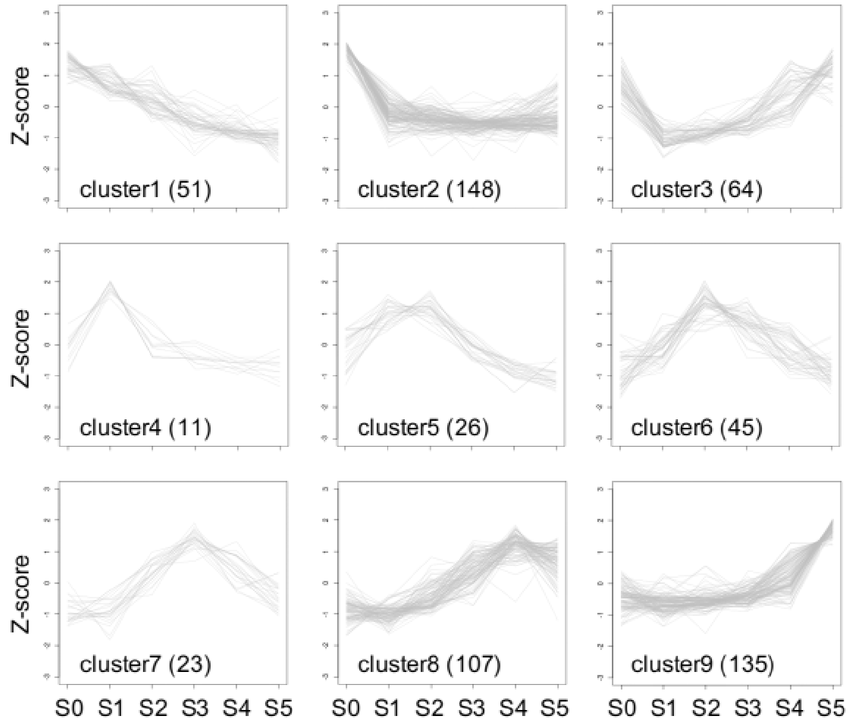

Fig. H. Coexpression network analysis for XUTs. Among 762 XUTs at S0, we identified 638 transcripts that were differentially expressed in at least one developmental stage at 5% FDR. With the differentially expressed XUTs, we performed coexpression network analysis, as did for the 547 lncRNAs (see Methods). Trend plots of Z-score normalized expression values for XUTs (number in parenthesis) in a given cluster were presented. Other five clusters that have less than 11 members were not shown. Note that the expression of clusters 8 and 9 showed increasing patterns across the perithecia development, peaking at S4 or S5.

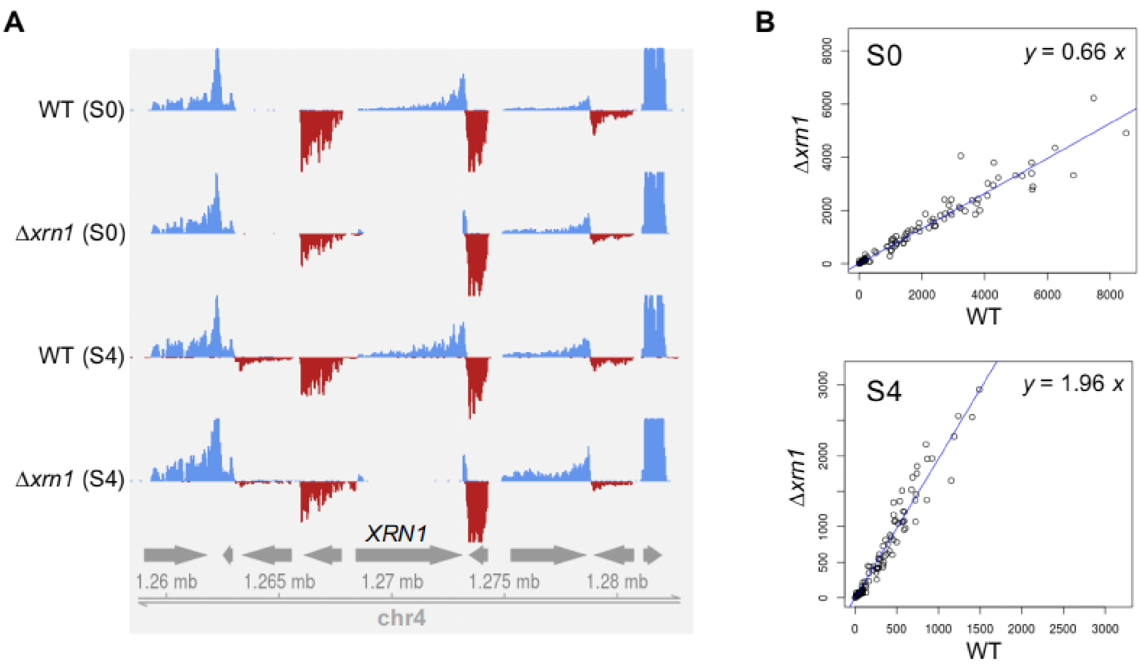

Fig. I. Transcriptome data comparison for the wild-type and Δ*xrn1*. (*A*) Visualization of transcriptome data on the *XRN1* locus before the normalization with the expression levels of ribosomal protein gene. Per-base coverage of transcripts was plotted for both DNA strands. Mapped reads of 3 biological replicates were pooled, then subsampled to 45 million reads for visual comparison of expression levels between the wild-type (WT) and Δ*xrn1* strain at S0 and S4. Note that no RNA-seq reads was mapped to the *XRN1* gene locus in Δ*xrn1*, confirming the targeted gene replacement and homokaryotic nuclei status. (*B*) For the normalization of dataset, ribosomal protein gene annotations were retrieved from the Munich Information Center for Protein Sequences (MIPS) *F. graminearum* genome database (v3.2; http://bioinformatik.wzw.tum.de), of which 124 genes showed expression in our dataset. The RPKM expression values for 124 ribosomal protein genes between WT (ordinate of plots) and Δ*xrn1* strain (abscissa of plots) were displayed in plots for transcriptome data at S0 and S4. Blue lines figure the regression lines with intersection fixed to zero.

Table A. Functional enrichment analyses for differentially expressed genes between two successive developmental stages.

			S0 vs. S1		S1 vs. S2		S2 vs. S3		S3 vs. S4		S4 vs. S5	
**Gene Ontology**	**Functional Term**	**Total[a]**	**DE[b]**	**adj. *p*-value**	**DE**	**adj. *p*-value**	**DE**	**adj. *p*-value**	**DE**	**adj. *p*-value**	**DE**	**adj. *p*-value**
GO:0000070	mitotic sister chromatid segregation	46	0	1.0000	0	1.0000	0	1.0000	0	1.0000	0	1.0000
GO:0005975	carbohydrate metabolic process	648	5	0.1082	1	0.5829	6	0.0073	3	0.1948	2	0.5686
GO:0006091	generation of precursor metabolites and energy	88	1	0.2890	0	1.0000	0	1.0000	0	1.0000	0	1.0000
GO:0006259	DNA metabolic process	360	0	1.0000	0	1.0000	0	1.0000	2	0.2100	0	1.0000
GO:0006260	DNA replication	72	0	1.0000	0	1.0000	0	1.0000	0	1.0000	0	1.0000
GO:0006281	DNA repair	245	0	1.0000	0	1.0000	0	1.0000	1	0.4486	0	1.0000
GO:0006310	DNA recombination	114	0	1.0000	0	1.0000	0	1.0000	2	0.0292	0	1.0000
GO:0006325	chromatin organization	222	1	0.5957	1	0.2526	0	1.0000	0	1.0000	0	1.0000
GO:0006351	transcription, DNA-templated	735	1	0.9572	1	0.6387	0	1.0000	0	1.0000	0	1.0000
GO:0006355	regulation of transcription, DNA-templated	937	1	0.9833	1	0.7335	0	1.0000	1	0.9108	1	0.9481
GO:0006412	translation	237	0	1.0000	1	0.2415	0	1.0000	0	1.0000	0	1.0000
GO:0006457	protein folding	113	0	1.0000	0	1.0000	0	1.0000	0	1.0000	0	1.0000
GO:0006461	protein complex assembly	211	0	1.0000	0	1.0000	0	1.0000	0	1.0000	0	1.0000
GO:0006464	cellular protein modification process	663	3	0.5020	1	0.5931	2	0.5791	1	0.8116	2	0.5832
GO:0006468	protein phosphorylation	146	1	0.4510	0	1.0000	0	1.0000	1	0.2967	1	0.3524
GO:0006520	cellular amino acid metabolic process	402	2	0.4848	1	0.4151	1	0.6954	3	0.0661	2	0.3254
GO:0006629	lipid metabolic process	610	7	0.0081	1	0.5619	1	0.8405	0	1.0000	3	0.2544
GO:0006766	vitamin metabolic process	60	1	0.2090	0	1.0000	0	1.0000	0	1.0000	0	1.0000
GO:0006865	amino acid transport	114	1	0.3728	0	1.0000	0	1.0000	2	0.0292	1	0.2866
GO:0006869	lipid transport	89	0	1.0000	0	1.0000	1	0.2261	0	1.0000	1	0.2254
GO:0006914	autophagy	66	0	1.0000	0	1.0000	0	1.0000	0	1.0000	0	1.0000
GO:0006950	response to stress	731	2	0.8057	1	0.6294	2	0.6350	1	0.8430	1	0.8927
GO:0006996	organelle organization	802	1	0.9665	1	0.6613	0	1.0000	4	0.1131	0	1.0000
GO:0007049	cell cycle	158	0	1.0000	0	1.0000	1	0.3674	0	1.0000	0	1.0000
GO:0007155	cell adhesion	74	0	1.0000	0	1.0000	1	0.1920	0	1.0000	0	1.0000
GO:0007165	signal transduction	426	0	1.0000	0	1.0000	0	1.0000	1	0.6504	2	0.3524
GO:0008643	carbohydrate transport	108	0	1.0000	0	1.0000	0	1.0000	0	1.0000	1	0.2756
GO:0009056	catabolic process	1,131	9	0.0247	1	0.7953	2	0.8600	3	0.5213	3	0.6528
GO:0010608	posttranscriptional regulation of gene expression	135	0	1.0000	0	1.0000	0	1.0000	0	1.0000	0	1.0000
GO:0016070	RNA metabolic process	1,327	1	0.9975	1	0.8535	0	1.0000	0	1.0000	1	0.9866
GO:0016192	vesicle-mediated transport	320	0	1.0000	0	1.0000	0	1.0000	0	1.0000	1	0.6098

Table A. (continued)

Gene Ontology	Functional Term	Total[a]	S0 vs. S1		S1 vs. S2		S2 vs. S3		S3 vs. S4		S4 vs. S5	
			DE[b]	adj. *p*-value	DE	adj. *p*-value	DE	adj. *p*-value	DE	adj. *p*-value	DE	adj. *p*-value
GO:0016570	histone modification	98	1	0.3277	1	0.1201	0	1.0000	0	1.0000	0	1.0000
GO:0019725	cellular homeostasis	204	2	0.1931	1	0.2315	1	0.4473	1	0.3899	0	1.0000
GO:0019748	secondary metabolic process	164	4	0.0037	1	0.1906	1	0.3783	0	1.0000	0	1.0000
GO:0022613	ribonucleoprotein complex biogenesis	85	0	1.0000	0	1.0000	0	1.0000	0	1.0000	0	1.0000
GO:0030163	protein catabolic process	124	0	1.0000	0	1.0000	0	1.0000	0	1.0000	0	1.0000
GO:0030437	ascospore formation	25	0	1.0000	0	1.0000	0	1.0000	2	0.0015	0	1.0000
GO:0032502	developmental process	429	1	0.8325	0	1.0000	2	0.3496	2	0.2721	4	0.0317
GO:0034293	sexual sporulation	30	0	1.0000	0	1.0000	0	1.0000	2	0.0022	0	1.0000
GO:0051169	nuclear transport	76	0	1.0000	0	1.0000	0	1.0000	0	1.0000	0	1.0000
GO:0051186	cofactor metabolic process	228	1	0.6024	0	1.0000	1	0.4853	2	0.1005	0	1.0000
GO:0051276	chromosome organization	170	0	1.0000	0	1.0000	0	1.0000	3	0.0069	0	1.0000
GO:0051301	cell division	161	0	1.0000	0	1.0000	0	1.0000	1	0.3220	0	1.0000
GO:0051726	regulation of cell cycle	265	1	0.6631	0	1.0000	0	1.0000	0	1.0000	0	1.0000
GO:0055085	transmembrane transport	852	2	0.8753	2	0.3053	2	0.7216	3	0.3309	3	0.4584
GO:0055086	nucleobase-containing small molecule metabolism	282	1	0.6793	0	1.0000	1	0.5616	2	0.1430	3	0.0428
GO:0061024	membrane organization	216	0	1.0000	0	1.0000	0	1.0000	1	0.4076	0	1.0000
GO:0070647	protein modification	180	0	1.0000	0	1.0000	0	1.0000	0	1.0000	1	0.4083
GO:0071554	cell wall organization or biogenesis	170	1	0.4968	0	1.0000	3	0.0117	2	0.0603	1	0.3902
GO:0071941	nitrogen cycle metabolic process	40	0	1.0000	0	1.0000	0	1.0000	0	1.0000	0	1.0000
GO:1901135	carbohydrate derivative metabolic process	341	1	0.7509	0	1.0000	5	0.0021	1	0.5662	4	0.0139
GO:1901990	regulation of mitotic cell cycle phase transition	101	0	1.0000	0	1.0000	0	1.0000	0	1.0000	0	1.0000
GO:1903046	meiotic cell cycle process	132	0	1.0000	0	1.0000	1	0.3173	4	0.0002	0	1.0000

[a] The total number of genes assigned to each GO term

[b] The number of differentially expressed genes

Table B. Conserved lncRNAs in other eukaryotes.

Antisense lncRNA			Sense mRNA		
F. graminearum	Conserved lncRNAs[a]	*E*-value	*F. graminearum*	Orthologs[b]	Description (similarity[c])
lncRNA-065	URS0000238E93 (*S. pombe*)	2.3e-29	FGRRES_01336	SPBC29A10.15	origin recognition complex subunit 1 (45%)
lncRNA-196	URS00000A491C (*S. pombe*)	6.2e-25	FGRRES_08458	SPAC16C9.02c	multicopy enhancer of UAS2 (53%)
lncRNA-303	URS0000807F52 (*D. melanogaster*)	3.7e-39	FGRRES_04922	FBgn0022359	sorbitol dehydrogenase (47%)
lncRNA-324	URS00003437F6 (*S. pombe*)	1e-13	FGRRES_05344	SPBC31F10.13c	histone regulatory protein 1 (40%)
lncRNA-477	URS00002B9743 (*S. pombe*)	1.4e-26	FGRRES_07582	SPCC1235.13	hexose transmembrane transporter (41%)

[a] RNAcentral database ID of fission yeast and *Drosophila melanogaster* lncRNAs.

[b] PomBase or FlyBase ID of orthologous genes overlapped to the conserved lncRNAs on the opposite strand.

[c] Similarity of deduced amino acids sequence of sense mRNAs between *F. graminearum* and other eukaryotes.

Table C. Expression correlation of selected lncRNAs and neighboring genes.

Orientation (*locus*)	Tandem (*orc1*)		Tandem (*orc2*)		Tandem (*cenp-T*)		Divergent (*hir1*)		Divergent (*nse4*)		Convergent (*cenp-T*)		Convergent (*rmd1*)	
Gene ID	lncRNA-065	FGRRES_01337	lncRNA-356	FGRRES_06123	lncRNA-235	FGRRES_08860	lncRNA-324	FGRRES_05345	lncRNA-201	FGRRES_13350	lncRNA-235	FGRRES_08858	lncRNA-430	FGRRES_06758
Sample 1[a]	0.0[b]	53.6	0.0	21.1	0.2	55.3	0.0	0.0	17.8	220.7	0.2	33.6	0.0	18.0
Sample 2	0.0	33.9	0.1	15.9	0.1	48.3	0.0	0.0	8.5	84.2	0.1	22.8	0.0	11.3
Sample 3	0.0	43.4	0.0	16.4	0.0	59.9	0.0	0.0	19.6	204.8	0.0	29.6	0.1	15.6
Sample 4	0.0	27.3	0.0	11.4	0.2	43.0	0.0	0.2	16.9	101.6	0.2	25.1	0.1	13.2
Sample 5	0.0	31.1	0.0	10.5	0.1	44.2	0.0	0.2	17.7	112.4	0.1	29.4	0.3	14.2
Sample 6	0.0	25.1	0.0	9.7	0.1	34.3	0.0	0.2	12.4	130.8	0.1	21.5	0.1	20.2
Sample 7	0.2	33.1	0.0	12.2	0.6	35.9	0.0	0.6	14.6	131.5	0.6	40.0	0.4	17.2
Sample 8	0.0	30.8	0.2	8.8	4.4	41.9	0.0	2.0	16.6	118.9	4.4	28.4	1.8	22.8
Sample 9	0.3	26.5	0.1	9.2	4.9	28.7	0.2	1.6	8.8	79.7	4.9	20.9	2.4	8.5
Sample 10	0.9	30.4	0.4	11.2	35.0	30.7	2.0	20.3	10.6	74.0	35.0	24.7	7.5	19.5
Sample 11	0.7	26.7	0.7	10.7	34.7	37.2	2.6	11.9	9.9	67.4	34.7	20.6	9.7	7.0
Sample 12	1.1	27.1	1.5	10.4	55.2	27.9	7.4	22.8	6.9	46.7	55.2	22.8	11.4	14.2
Sample 13	2.7	27.7	2.6	12.8	33.0	28.4	8.0	74.2	8.7	37.4	33.0	19.5	19.3	7.0
Sample 14	2.8	28.7	2.6	13.4	31.7	22.4	10.8	138.5	5.7	34.6	31.7	15.1	16.7	10.2
Sample 15	2.9	32.1	2.1	15.6	34.6	26.0	7.4	96.1	4.8	37.3	34.6	15.1	17.2	17.6
Sample 16	2.0	28.2	2.5	19.9	2.5	28.2	3.6	104.1	4.1	54.5	2.5	17.2	9.8	8.5
Sample 17	2.5	27.2	4.8	19.2	5.2	21.8	1.2	52.4	3.0	43.7	5.2	14.6	12.3	8.2
Sample 18	1.3	29.3	2.2	21.8	2.0	28.0	2.3	59.7	2.9	58.4	2.0	16.7	5.9	10.1
Correlation[c]	-0.32		0.47		-0.49		0.82		0.84		-0.36		-0.44	

[a] perithecia transcriptome dataset (Samples 1–18) was rearranged by the BLIND program (Fig. B).

[b] Gene expression levels were represented in RPKM.

[c] Pearson's correlation coefficient between lncRNA expression and its neighboring gene expression.

Table D. Top 80 sRNA clusters across the *F. graminearum* genome**.**

Cluster ID	Coordinate	Mapped reads	Coding gene IDs[a]	Noncoding gene IDs[b]	Class[c]
Cluster_1039	1:5657102-5660576	124215	FGRRES_01706	EFFGRG00000014717	tRNA
Cluster_77	1:677771-682182	110071	FGRRES_11711	.	.
Cluster_5248	4:438145-442308	100446	4:440584-442254(-)	lncRNA-416	lncRNA
Cluster_2191	1:11073393-11077776	94480	FGRRES_10502	lncRNA-553	lncRNA
Cluster_2873	2:2987800-2991657	93364	FGRRES_15524	.	.
Cluster_2361	2:863882-867011	84498	2:864911-865686(-)	.	.
Cluster_3481	2:6848497-6852151	79385	FGRRES_16156	.	.
Cluster_6710	4:7884555-7888050	79134	FGRRES_13494	lncRNA-545	lncRNA
Cluster_937	1:5021680-5024969	61312	FGRRES_01518	EFFGRG00000014656	tRNA
Cluster_2829	2:2833777-2834797	58605	FGRRES_08833	.	.
Cluster_1595	1:8430563-8433719	57199	FGRRES_02620_M	.	.
Cluster_2388	2:954036-957627	55081	FGRRES_08301_M	.	.
Cluster_1356	1:7131145-7134627	51581	FGRRES_15946_M	.	.
Cluster_1223	1:6597990-6601098	50155	FGRRES_15015_M	EFFGRG00000014573	tRNA
Cluster_6594	4:7331045-7333722	49444	FGRRES_09193	FGRRES_ncRNA013482	tRNA
Cluster_3743	2:8216261-8220177	48941	FGRRES_20213	.	.
Cluster_5409	4:1089874-1092276	47868	FGRRES_12982	FGRRES_ncRNA013470	tRNA
Cluster_3998	3:983398-985455	46655	FGRRES_17642	.	.
Cluster_2076	1:10852150-10854716	46166	FGRRES_13756	lncRNA-174	lncRNA
Cluster_3993	3:947385-951823	43928	FGRRES_05031	.	.
Cluster_1621	1:8602176-8606680	41441	FGRRES_02677	.	.
Cluster_2133	1:10986730-10987553	39465	FGRRES_15605_M	.	.
Cluster_2132	1:10985731-10986714	39389	FGRRES_13764	.	.
Cluster_231	1:1486645-1486825	39090	1:1486174-1486676(+)	FGRRES_ncRNA013801	U2-snRNA
Cluster_5914	4:3931354-3934344	38963	FGRRES_15436	lncRNA-477	lncRNA
Cluster_2359	2:862930-863721	38727	FGRRES_08268	.	.
Cluster_3163	2:4550729-4553792	38337	FGRRES_16307_M	lncRNA-250	lncRNA
Cluster_5032	3:6585477-6587120	37902	FGRRES_11086	.	.
Cluster_5932	4:3994203-3995647	35941	FGRRES_07605	.	.
Cluster_3786	2:8372834-8373780	35274	FGRRES_04550_M	.	.
Cluster_5002	3:6452461-6455811	34872	FGRRES_13867	.	.
Cluster_3338	2:6074670-6076504	34048	FGRRES_03793	.	.
Cluster_1669	1:8894273-8896051	33806	FGRRES_12198	.	.
Cluster_39	1:515176-516965	33484	FGRRES_00156_M	lncRNA-548	lncRNA
Cluster_3980	3:877398-880583	32318	FGRRES_12667	lncRNA-556	lncRNA
Cluster_2293	2:209832-212343	31417	FGRRES_13452	.	.
Cluster_2411	2:1039709-1042568	30821	2:1039318-1041402(-)	.	.
Cluster_2072	1:10826029-10827359	30017	FGRRES_17358	.	.
Cluster_3685	2:7978418-7980743	27375	2:7978596-7981095(+)	lncRNA-290	lncRNA
Cluster_1516	1:8001451-8003334	27151	FGRRES_02486	lncRNA-551	lncRNA
Cluster_6644	4:7418464-7419120	26830	FGRRES_13523	lncRNA-536	lncRNA
Cluster_2484	2:1192947-1195909	26341	FGRRES_08374	EFFGRG00000014522	tRNA
Cluster_3501	2:6872686-6875803	26224	FGRRES_12310	lncRNA-555	lncRNA
Cluster_3763	2:8334546-8337964	25991	FGRRES_04531	.	.

Table D. (continued)

Cluster ID	Coordinate	Mapped reads	Coding gene IDs[a]	Noncoding gene IDs[b]	Class[c]
Cluster_2835	2:2837145-2839849	25884	FGRRES_08835	EFFGRG00000014737	tRNA
Cluster_931	1:5013298-5018208	25806	1:5014599-5017422(-)	.	.
Cluster_6701	4:7842823-7845570	25605	FGRRES_09006_M	.	.
Cluster_2158	1:11010210-11017715	25500	FGRRES_10483	lncRNA-552	lncRNA
Cluster_3942	3:806786-807550	25419	FGRRES_16447	lncRNA-557	lncRNA
Cluster_5163	3:7414157-7417048	23884	FGRRES_11380_M	.	.
Cluster_2878	2:2994940-2997335	23636	FGRRES_08884	.	.
Cluster_4633	3:4291117-4293424	23011	FGRRES_06095	FGRRES_ncRNA014090	U4-snRNA
Cluster_4710	3:4656796-4659145	22555	FGRRES_12891_M	.	.
Cluster_250	1:1563616-1566440	22284	FGRRES_11773	.	.
Cluster_1481	1:7830010-7831363	22098	FGRRES_02429	.	.
Cluster_1506	1:7923106-7925456	21907	FGRRES_02465	.	.
Cluster_3017	2:3715376-3718061	20791	FGRRES_12556_M	.	.
Cluster_6738	Mt:10-3164	20672	Mt:2085-5369(+)	.	.
Cluster_3742	2:8213963-8216229	19747	FGRRES_16106	.	.
Cluster_5695	4:2670919-2673580	18979	FGRRES_07187_M	.	.
Cluster_6039	4:4457122-4459092	18741	FGRRES_20378	.	.
Cluster_5033	3:6587170-6589055	18378	FGRRES_11087	.	.
Cluster_100	1:808124-809995	17917	FGRRES_00255	.	.
Cluster_6144	4:4990329-4990555	17862	FGRRES_07949	FGRRES_ncRNA013328	U5-snRNA
Cluster_4529	3:3824188-3826717	17680	FGRRES_15324	.	.
Cluster_5787	4:3254095-3256576	17505	FGRRES_07353_M	.	.
Cluster_2649	2:1900887-1901076	17429	FGRRES_08579	.	.
Cluster_2418	2:1066930-1069395	17177	FGRRES_08332	.	.
Cluster_6266	4:5742551-5745569	17168	FGRRES_17253	lncRNA-504	lncRNA
Cluster_2553	2:1566252-1569061	17081	FGRRES_08483_M	.	.
Cluster_2987	2:3553439-3555708	16790	FGRRES_02841	.	.
Cluster_6498	4:6892298-6894984	16737	FGRRES_09333	lncRNA-526	lncRNA
Cluster_391	1:2177981-2182076	16469	FGRRES_15768_M	lncRNA-549	lncRNA
Cluster_1360	1:7143259-7145495	16445	FGRRES_02194	lncRNA-550	lncRNA
Cluster_3291	2:5630573-5633174	16367	FGRRES_03621	.	.
Cluster_1272	1:6665363-6668177	15420	FGRRES_12061	FGRRES_ncRNA013803	tRNA
Cluster_2321	2:496897-498926	15339	FGRRES_20117	.	.
Cluster_2466	2:1163247-1166009	15312	FGRRES_17050	.	.
Cluster_740	1:4002166-4003938	15172	FGRRES_01214	.	.
Cluster_4447	3:3307765-3310421	15129	FGRRES_05761	.	.

[a] Listed were gene IDs of the closest coding genes to the center of respective sRNA clusters. For novel transcripts with CPAT score greater than 0.540 that were identified in this study, their genomic coordinates were provided.

[b] Gene IDs (Ensembl annotation v32) of noncoding genes and lncRNA IDs, if any, in sRNA clustres were presented.

[c] Noncoding genes were classified by Rfam database (v13.0) search (*E*-value < 10^{-10}; http://rfam.xfam.org).

Table E. Functional enrichment analyses for sense mRNAs that showed expression correlation with antisense lncRNAs.

Gene Ontology	Functional Term	Total[a]	DE[b]	adj. *p*-value
GO:0000070	mitotic sister chromatid segregation	46	0	0.0500
GO:0005975	carbohydrate metabolic process	648	3	0.5810
GO:0006091	generation of precursor metabolites and energy	88	0	1.0000
GO:0006259	DNA metabolic process	360	8	0.0004
GO:0006260	DNA replication	72	2	0.0561
GO:0006281	DNA repair	245	5	0.0092
GO:0006310	DNA recombination	114	2	0.1196
GO:0006325	chromatin organization	222	3	0.1133
GO:0006351	transcription, DNA-templated	735	3	0.7607
GO:0006355	regulation of transcription, DNA-templated	937	3	0.8902
GO:0006412	translation	237	0	1.0000
GO:0006457	protein folding	113	0	1.0000
GO:0006461	protein complex assembly	211	2	0.2398
GO:0006464	cellular protein modification process	663	4	0.4337
GO:0006468	protein phosphorylation	146	0	1.0000
GO:0006520	cellular amino acid metabolic process	402	0	1.0000
GO:0006629	lipid metabolic process	610	3	0.5491
GO:0006766	vitamin metabolic process	60	1	0.2208
GO:0006865	amino acid transport	114	0	1.0000
GO:0006869	lipid transport	89	0	1.0000
GO:0006914	autophagy	66	0	1.0000
GO:0006950	response to stress	731	6	0.1611
GO:0006996	organelle organization	802	4	0.5903
GO:0007049	cell cycle	158	2	0.1991
GO:0007155	cell adhesion	74	0	1.0000
GO:0007165	signal transduction	426	1	0.9151
GO:0008643	carbohydrate transport	108	1	0.3940

Table E. (continued)

Gene Ontology	Functional Term	Total[a]	DE[b]	adj. *p*-value
GO:0009056	catabolic process	1,131	6	0.4228
GO:0010608	posttranscriptional regulation of gene expression	135	0	1.0000
GO:0016070	RNA metabolic process	1,327	6	0.6918
GO:0016192	vesicle-mediated transport	320	1	0.8179
GO:0016570	histone modification	98	1	0.4028
GO:0019725	cellular homeostasis	204	1	0.6537
GO:0019748	secondary metabolic process	164	1	0.5859
GO:0022613	ribonucleoprotein complex biogenesis	85	0	1.0000
GO:0030163	protein catabolic process	124	2	0.1188
GO:0030437	ascospore formation	25	0	1.0000
GO:0032502	developmental process	429	1	0.9153
GO:0034293	sexual sporulation	30	0	1.0000
GO:0051169	nuclear transport	76	0	1.0000
GO:0051186	cofactor metabolic process	228	1	0.6621
GO:0051276	chromosome organization	170	0	1.0000
GO:0051301	cell division	161	2	0.2116
GO:0051726	regulation of cell cycle	265	3	0.1690
GO:0055085	transmembrane transport	852	4	0.5825
GO:0055086	nucleobase-containing small molecule metabolism	282	2	0.3755
GO:0061024	membrane organization	216	0	1.0000
GO:0070647	protein modification	180	1	0.6137
GO:0071554	cell wall organization or biogenesis	170	1	0.5613
GO:0071941	nitrogen cycle metabolic process	40	0	1.0000
GO:1901135	carbohydrate derivative metabolic process	341	3	0.2240
GO:1901990	regulation of mitotic cell cycle phase transition	101	3	0.0159
GO:1903046	meiotic cell cycle process	132	1	0.5115

[a] The total number of genes assigned to each GO term

[b] The number of differentially expressed genes

Table F. The annotations of additional lncRNAs found in sRNA clusters.

Cluster ID	lncRNA ID	Coordinate	Coding probability[a]	ORF[a]	Fickett score[a]	Best hit[b]	Similarity[b]	Query coverage[b]	*E*-value[b]
Cluster_39	lncRNA-548	1:514948-516144(-)	0.119	85	0.372	WP_083704694.1	33%	57%	9.9
Cluster_391	lncRNA-549	1:2178114-2180983(-)	0.314	137	0.363	No_hit	.	.	.
Cluster_1360	lncRNA-550	1:7143262-7144220(+)	0.175	132	0.355	WP_065955966.1	38%	47%	8.7
Cluster_1516	lncRNA-551	1:8001450-8003848(-)	0.401	158	0.297	OLC70875.1	32%	44%	3.7
Cluster_2158	lncRNA-552	1:11009596-11015742(-)	0.933	227	0.315	XP_009261170.1	81%	11%	6.0e-4
Cluster_2191	lncRNA-553	1:11075542-11077742(+)	0.196	89	0.398	OGU29779.1	40%	53%	9.6
Cluster_2418	lncRNA-554	2:1065605-1066500(-)	0.354	90	0.455	CCG84160.1	55%	34%	7.6
Cluster_3501	lncRNA-555	2:6875106-6876424(+)	0.339	136	0.331	KPA42729.1	57%	38%	3.0e-8
Cluster_3980	lncRNA-556	3:881278-884444(-)	0.081	93	0.301	No_hit	.	.	.
Cluster_3942	lncRNA-557	3:806961-807515(-)	0.035	49	0.396	KJB50059.1	45%	81%	8.3

[a] Coding probability, open reading frame (ORF) size (in amino acids), and Fickett score were computed by the CPC2 program (v2.0b; Kang et al. 2017).

[b] Protein BLAST results were reported for the deduced polypeptide sequences of putative lncRNAs in the NCBI website (searched on November 2017).

Literatures Cited

Anavy L, Levin M, Khair S, Nakanishi N, Fernandez-Valverde SL, Degnan BM, Yanai I. 2014. BLIND ordering of large-scale transcriptomic developmental timecourses. *Development* **141**: 1161–1166.

Anders S, Pyl PT, Huber W. 2015. HTSeq: a Python framework to work with high-throughput sequencing data. *Bioinformatics* **31**: 166–169.

Carroll AM, Sweigard JA, Valent B. 1994. Improved vectors for selecting resistance to hygromycin. Fungal Genet Newsl 41: 22.

Catlett NL, Lee B, Yoder OC, Turgeon BG. 2003. Split-marker recombination for efficient targeted deletion of fungal genes. Fungal Genet Newsl 50: 9–11.

Chen C, Khaleel SS, Huang H, Wu CH. 2014. Software for pre-processing Illumina next-generation sequencing short read sequences. *Source Code Biol Med* **9**: 8.

Finn RD, Coggill P, Eberhardt RY, Eddy SR, Mistry J, Mitchell AL, Potter SC, Punta M, Qureshi M, Sangrador-Vegas A, et al. 2016. The Pfam protein families database: towards a more sustainable future. *Nucleic Acids Res* **44**: D279–D285.

Frazee AC, Pertea G, Jaffe AE, Langmead B, Salzberg SL, Leek JT. 2015. Ballgown bridges the gap between transcriptome assembly and expression analysis. *Nat Biotechnol* **33**: 243–246.

Hallen-Adams HE, Cavinder BL, Trail F. 2011. *Fusarium graminearum* from expression analysis to functional assays. In Fungal Genomics, part of the Methods in Molecular Biology book series, vol 722. (ed. Xu JR, Bluhm B), pp. 79-101. Humana Press, New York.

Kang Y-J, Yang D-C, Kong L, Hou M, Meng Y-Q, Wei L, Gao G. 2017. CPC2: a fast and accurate coding potential calculator based on sequence intrinsic features. *Nucleic Acids Res* **45**: W12–W16.

Kim D, Langmead B, Salzberg SL. 2015. HISAT: a fast spliced aligner with low memory requirements. *Nat Methods* **12**: 357–360.

Nawrocki EP, Burge SW, Bateman A, Daub J, Eberhardt RY, Eddy SR, Floden EW, Gardner PP, Jones TA, Tate J, et al. 2015. Rfam 12.0: updates to the RNA families database. *Nucleic Acids Res* **43**: D130–D137.

Nawrocki EP, Eddy SR. 2013. Infernal 1.1: 100-fold faster RNA homology searches. *Bioinformatics* **29**: 2933–2935.

Pelechano V, Wei W, Steinmetz LM. 2013. Extensive transcriptional heterogeneity revealed by isoform profiling. *Nature* **497**: 127–131.

Pertea M, Kim D, Pertea GM, Leek JT, Salzberg SL. 2016. Transcript-level expression analysis of RNA-seq experiments with HISAT, StringTie and Ballgown. *Nat Protoc* **11**: 1650–1667.

Quinlan AR, Hall IM. 2010. BEDTools: a flexible suite of utilities for comparing genomic features. *Bioinformatics* **26**: 841–842.

Scotto–Lavino E, Du G, Frohman MA. 2006. 3′ End cDNA amplification using classic RACE. *Nat Protoc* **1**: 2742.

Son H, Park AR, Lim JY, Shin C, Lee Y-W. 2017. Genome-wide exonic small interference RNA-mediated gene silencing regulates sexual reproduction in the homothallic fungus *Fusarium graminearum*. *PLOS Genet* **13**: e1006595.

Teichert I, Wolff G, Kück U, Nowrousian M. 2012. Combining laser microdissection and RNA-seq to chart the transcriptional landscape of fungal development. *BMC Genomics* **13**: 511.

Wang L, Park HJ, Dasari S, Wang S, Kocher J-P, Li W. 2013. CPAT: Coding-Potential Assessment Tool using an alignment-free logistic regression model. *Nucleic Acids Res* **41**: e74.

Weirick T, Militello G, Müller R, John D, Dimmeler S, Uchida S. 2016. The identification and characterization of novel transcripts from RNA-seq data. *Brief Bioinform* **17**: 678–685.

Wheeler TJ, Eddy SR. 2013. nhmmer: DNA homology search with profile HMMs. *Bioinformatics* **29**: 2487–2489.

Yu J-H, Hamari Z, Han K-H, Seo J-A, Reyes-Domínguez Y, Scazzocchio C. 2004. Double-joint PCR: a PCR-based molecular tool for gene manipulations in filamentous fungi. *Fungal Genet Biol* **41**: 973–981.
