# Supplementary material for: Developmental Dynamics of Long Noncoding RNA Expression during Sexual Fruiting Body Formation in Fusarium graminearum
Source: mBio. 2018 Aug 14;9(4):e01292-18. doi: 10.1128/mBio.01292-18 (PMC6094484; doi:10.1128/mBio.01292-18)

Fig. S1. Visualization of the expression of lncRNAs associated with sRNA clusters

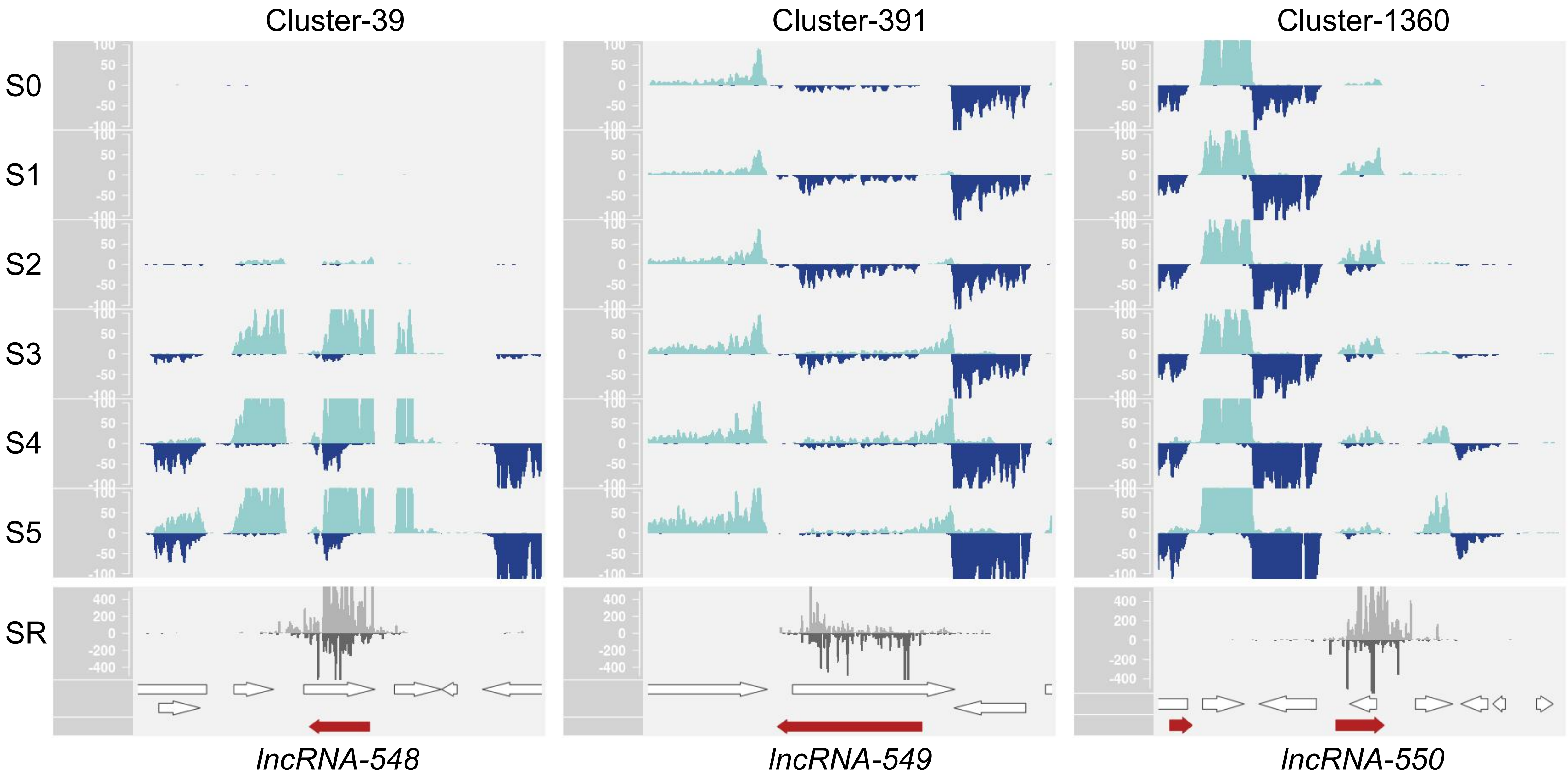

Fig. S1. (continued)

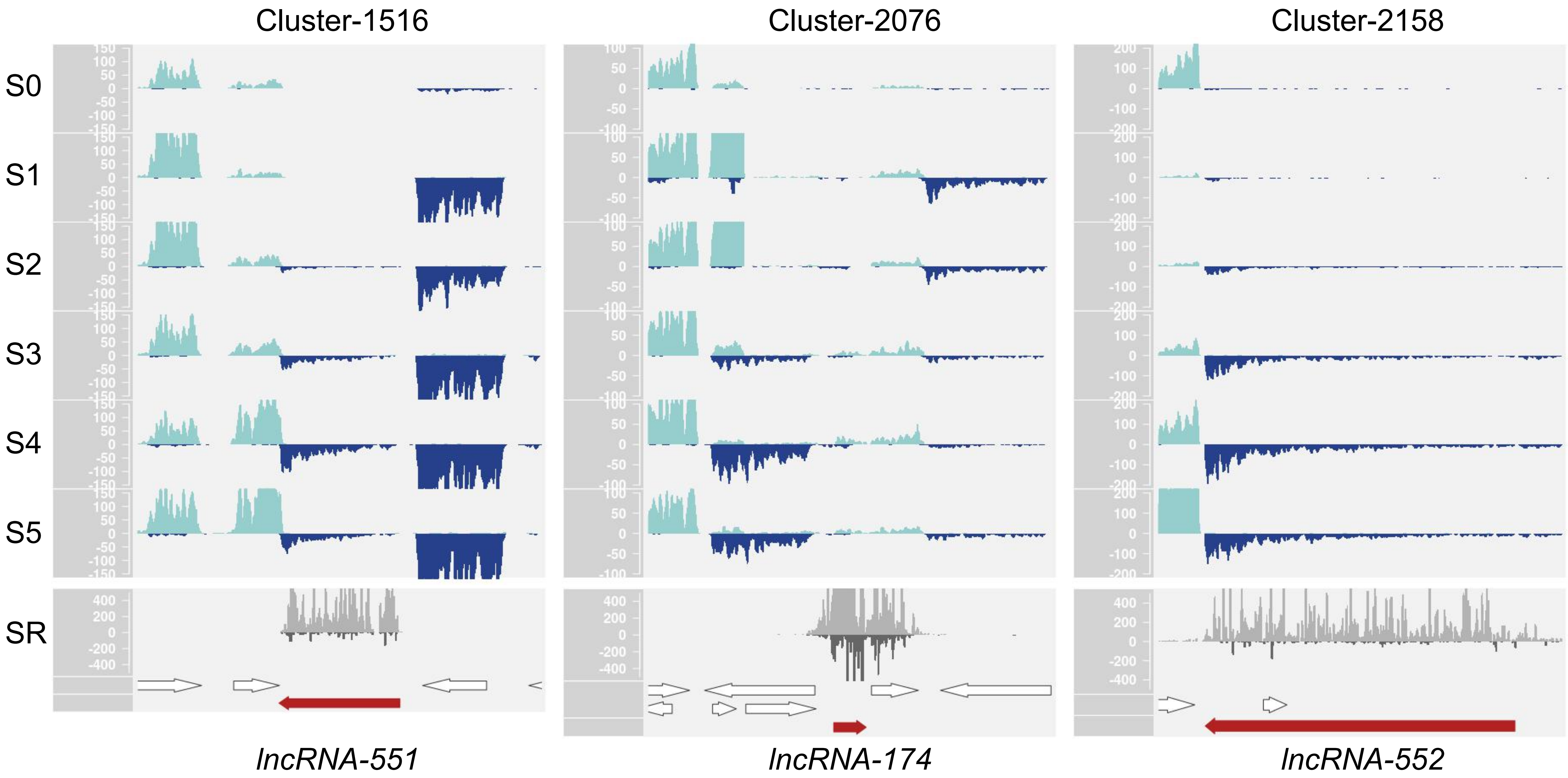

Fig. S1. (continued)

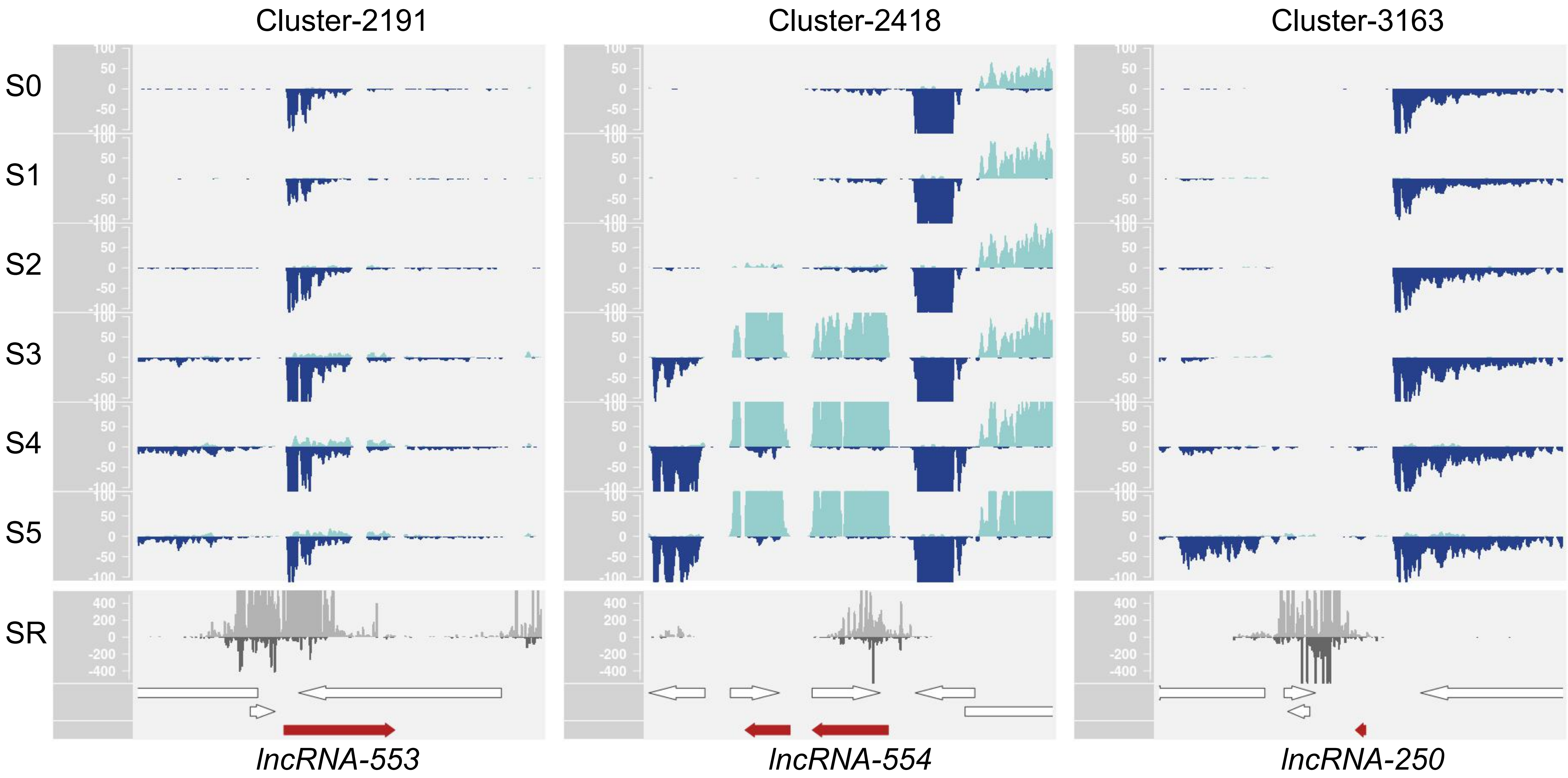

Fig. S1. (continued)

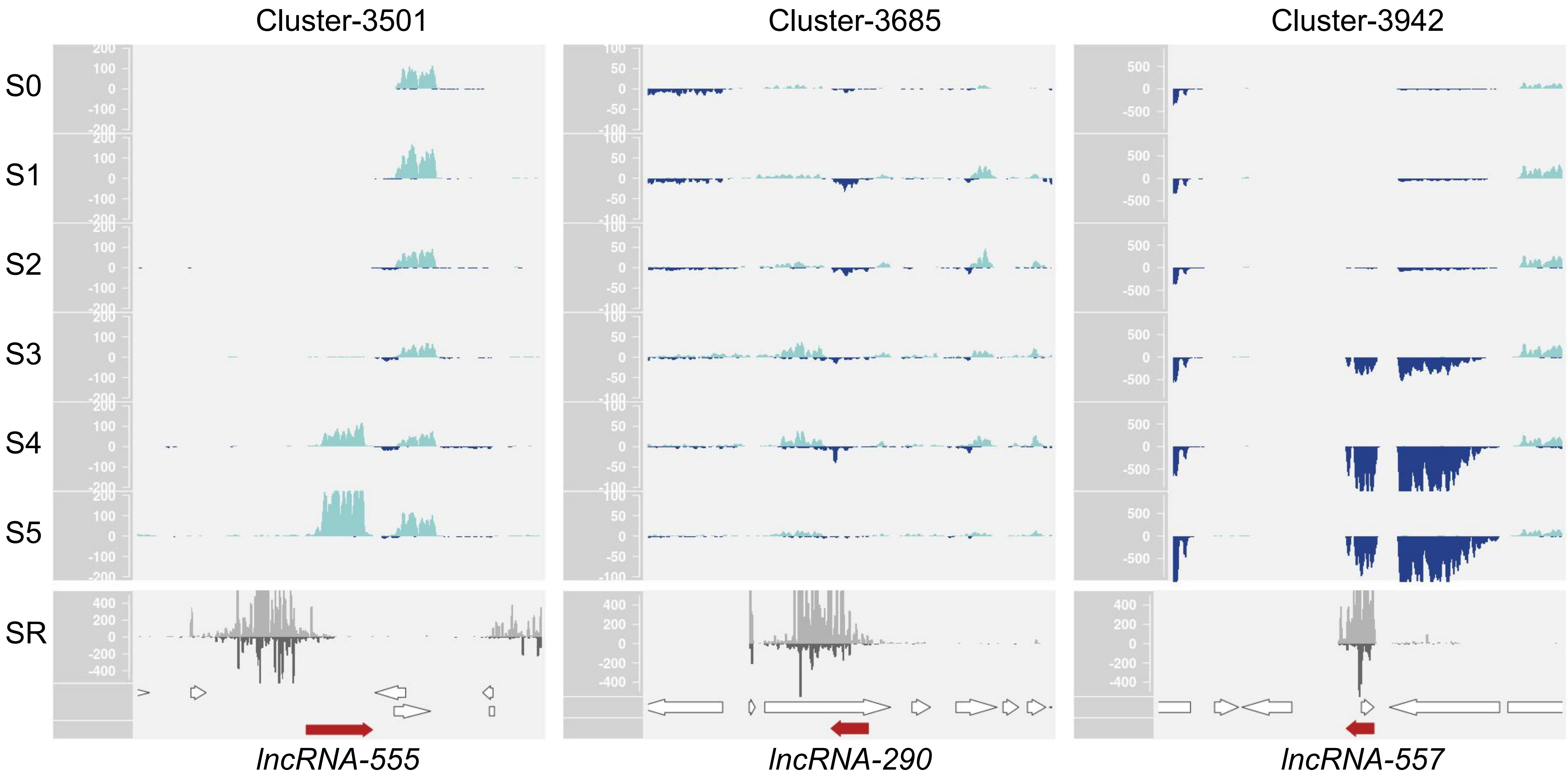

Fig. S1. (continued)

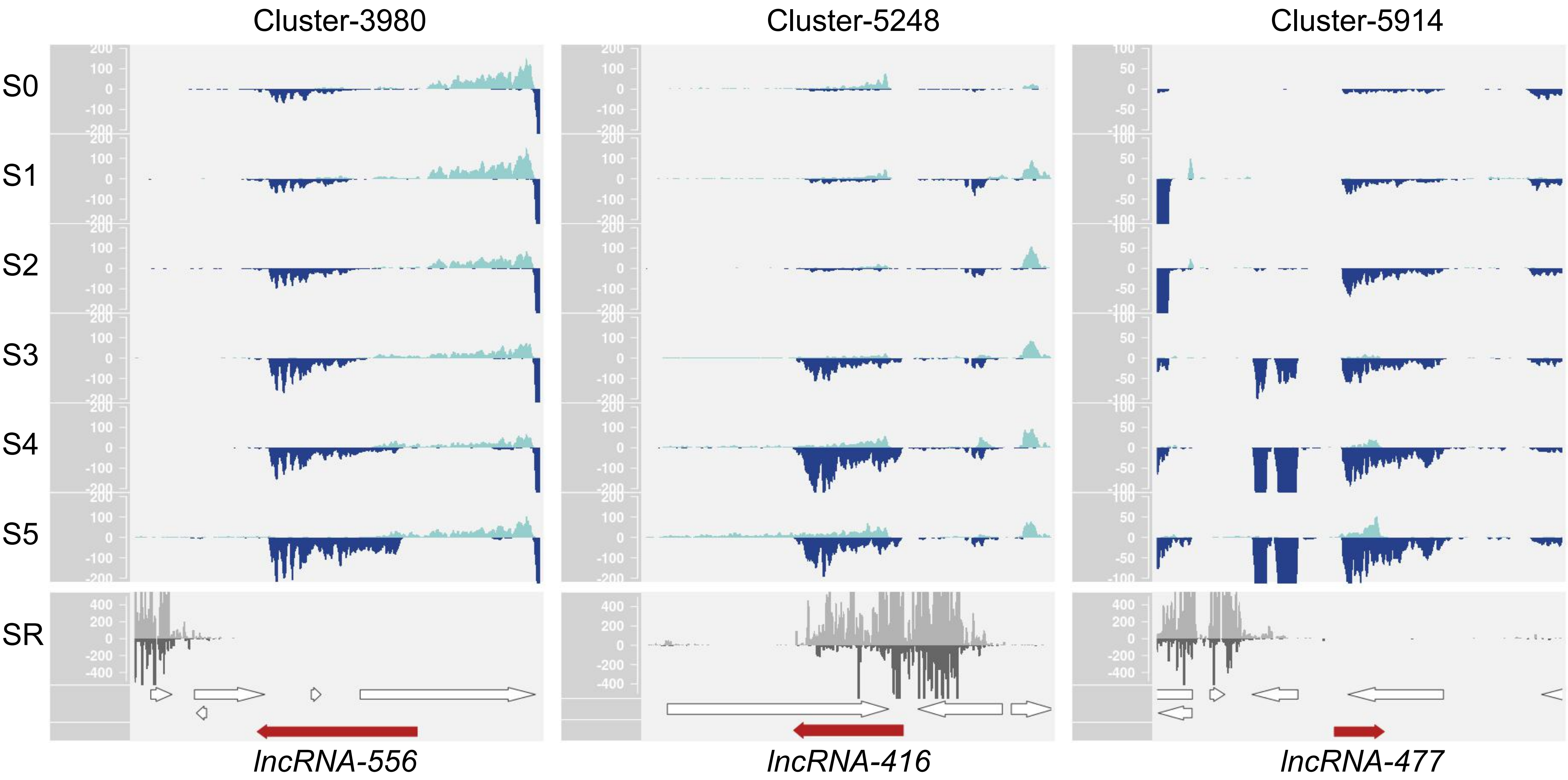

Fig. S1. (continued)

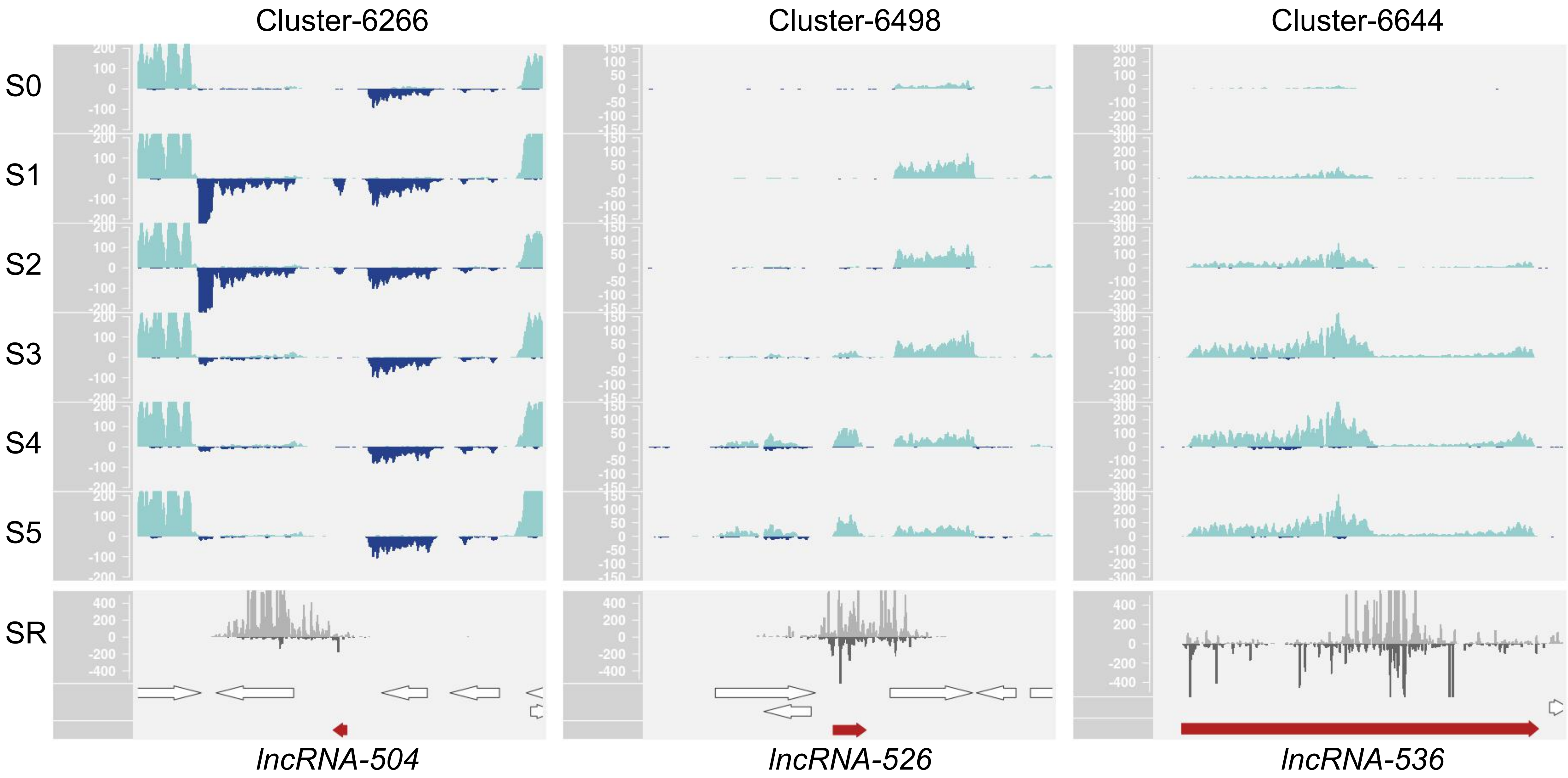

Fig. S1. (continued)

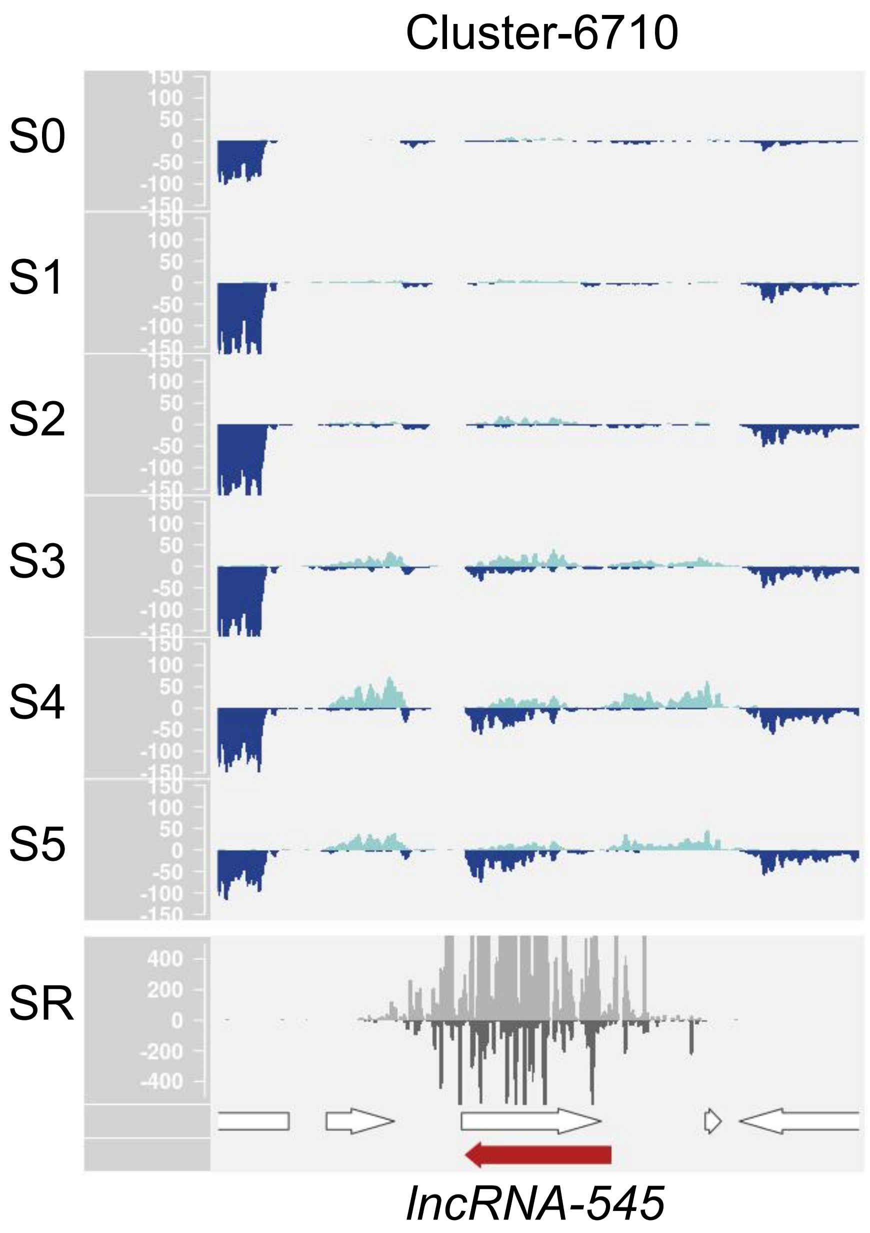

Supplement: FIG S1 [file mbo004184025sf1.pdf]
