# Supplementary material for: Developmental Dynamics of Long Noncoding RNA Expression during Sexual Fruiting Body Formation in Fusarium graminearum
Source: mBio. 2018 Aug 14;9(4):e01292-18. doi: 10.1128/mBio.01292-18 (PMC6094484; doi:10.1128/mBio.01292-18)
Supplement: FIG S2 [file mbo004184025sf2.pdf]

Fig. S2. Visualization of the expression of lncRNAs identified as XUTs

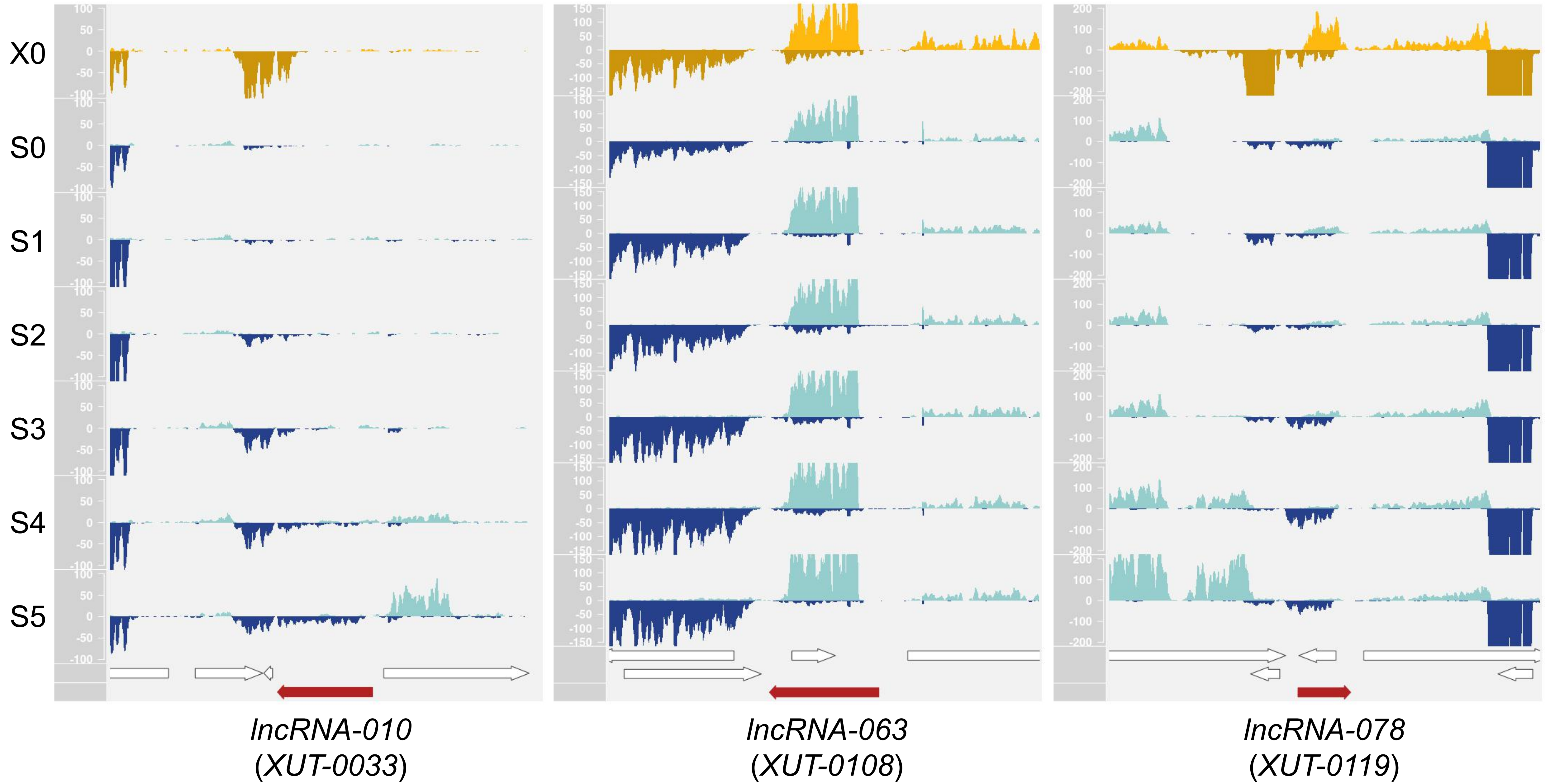

Fig. S2. (continued)

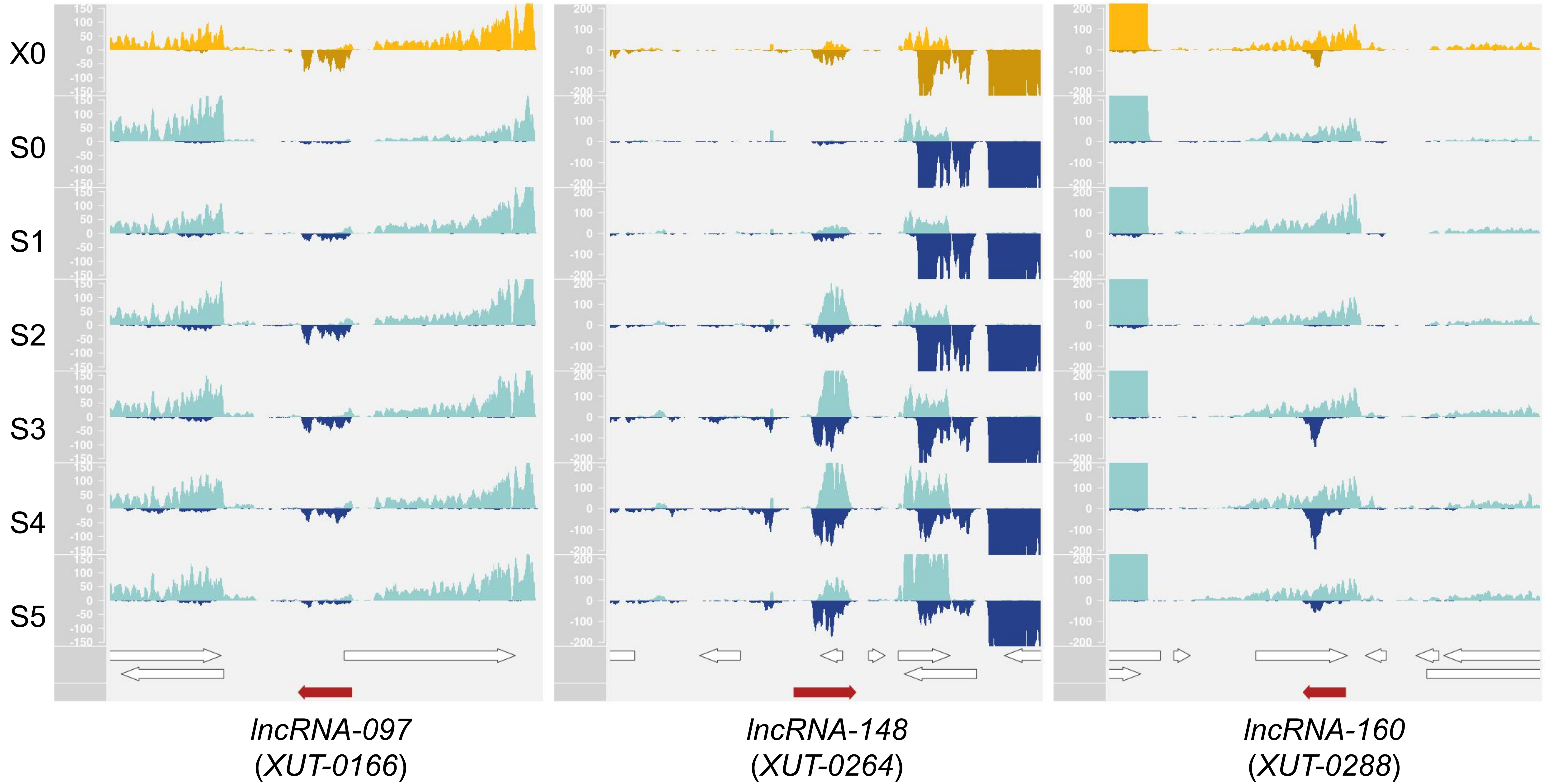

Fig. S2. (continued)

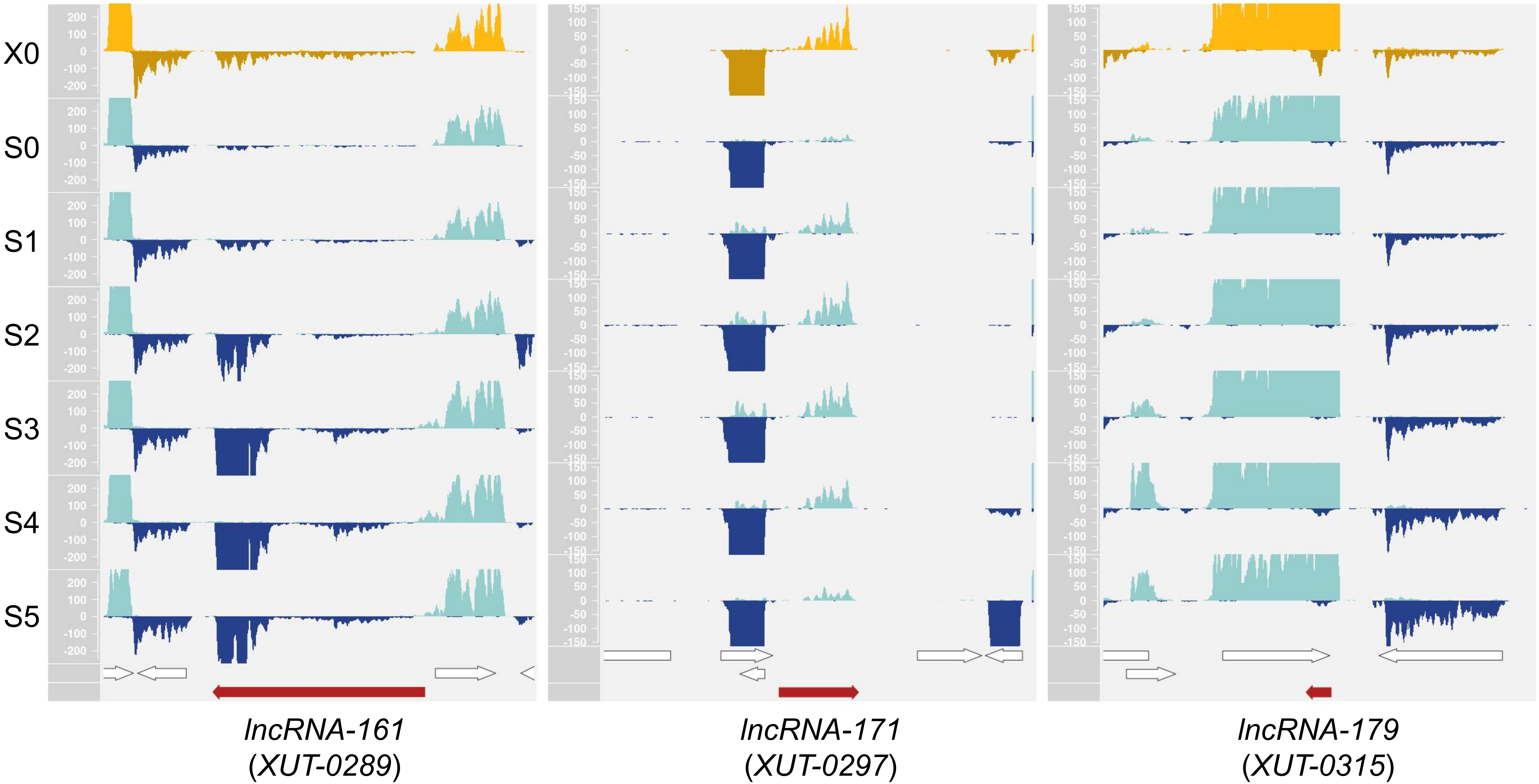

Fig. S2. (continued)

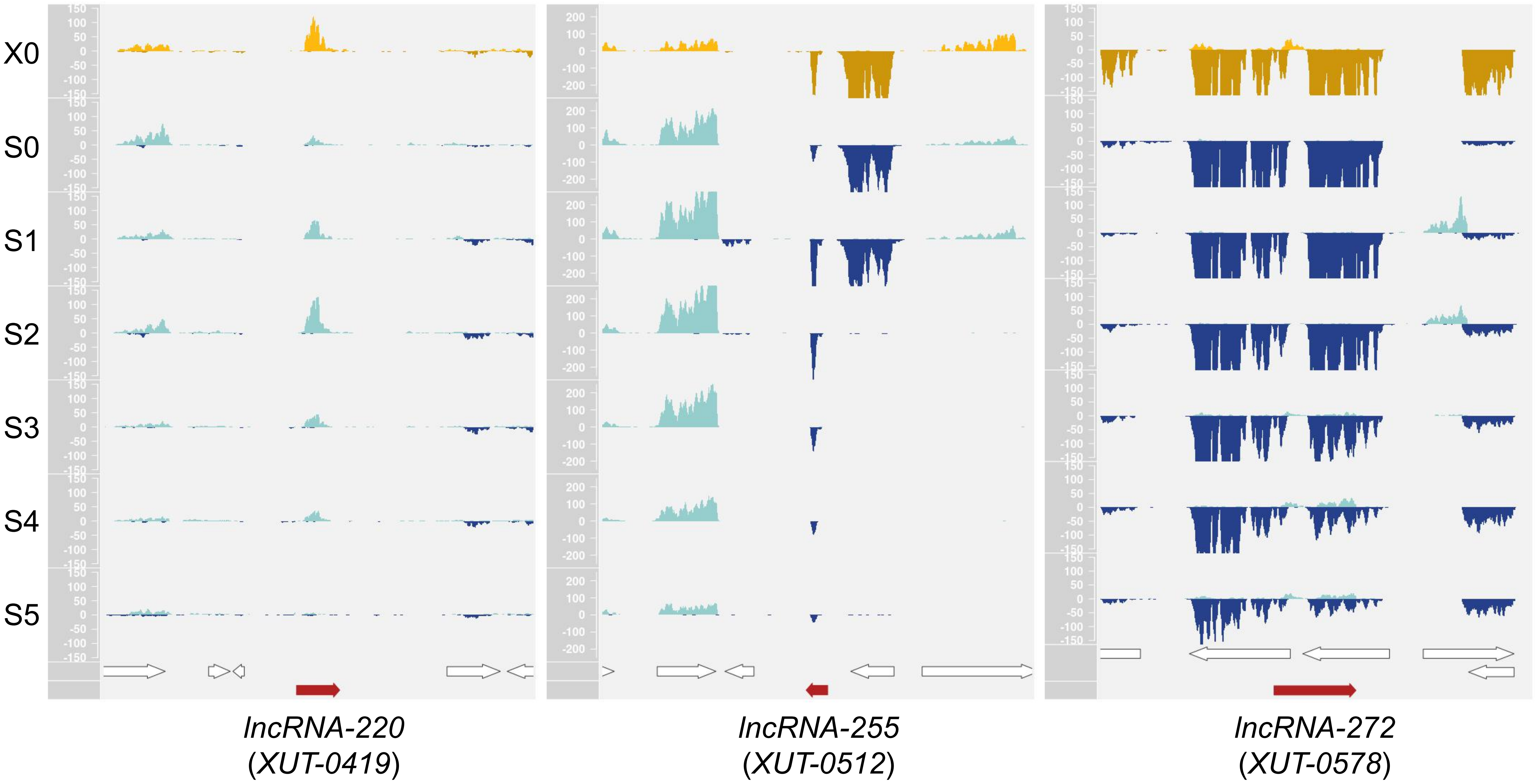

Fig. S2. (continued)

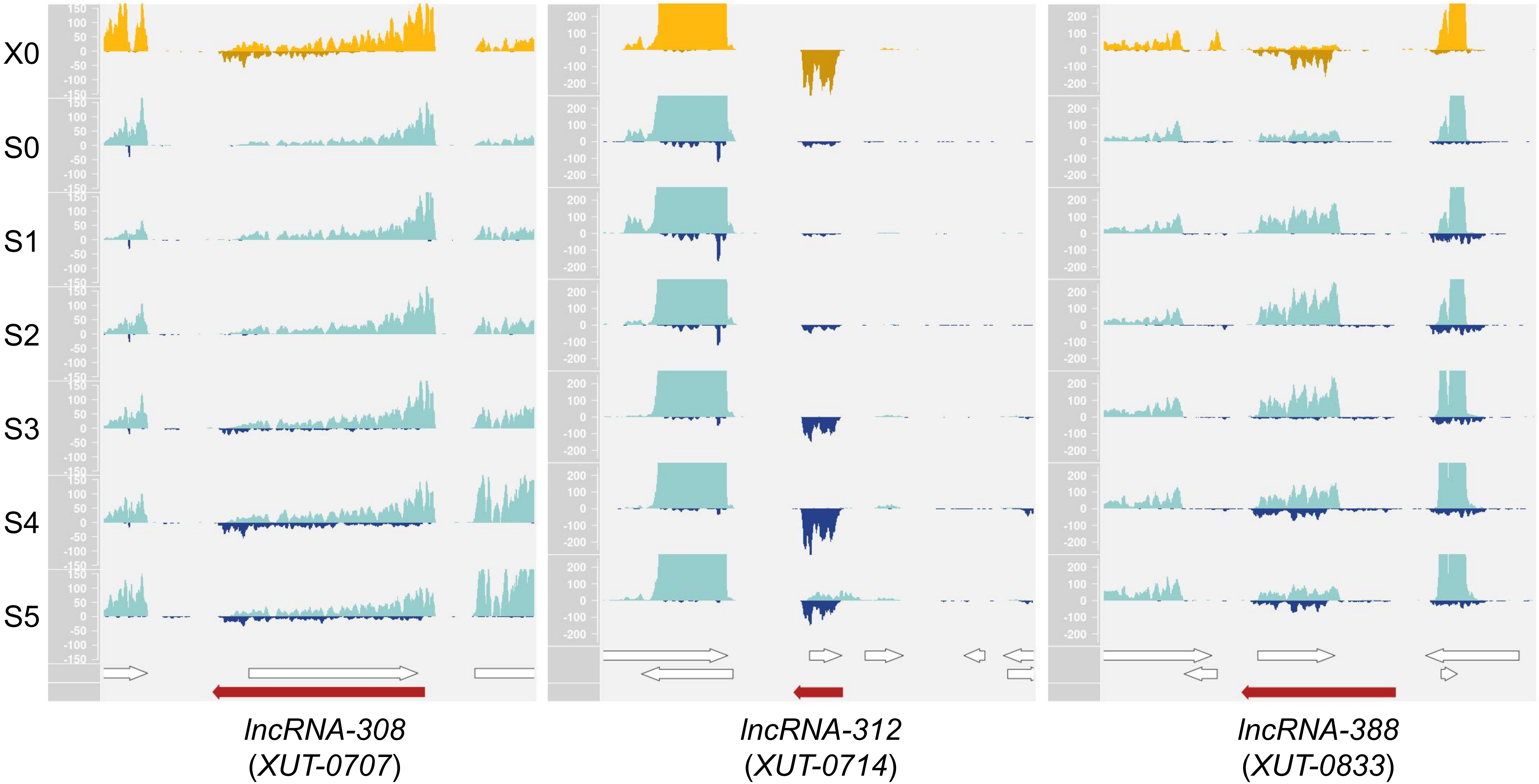

Fig. S2. (continued)

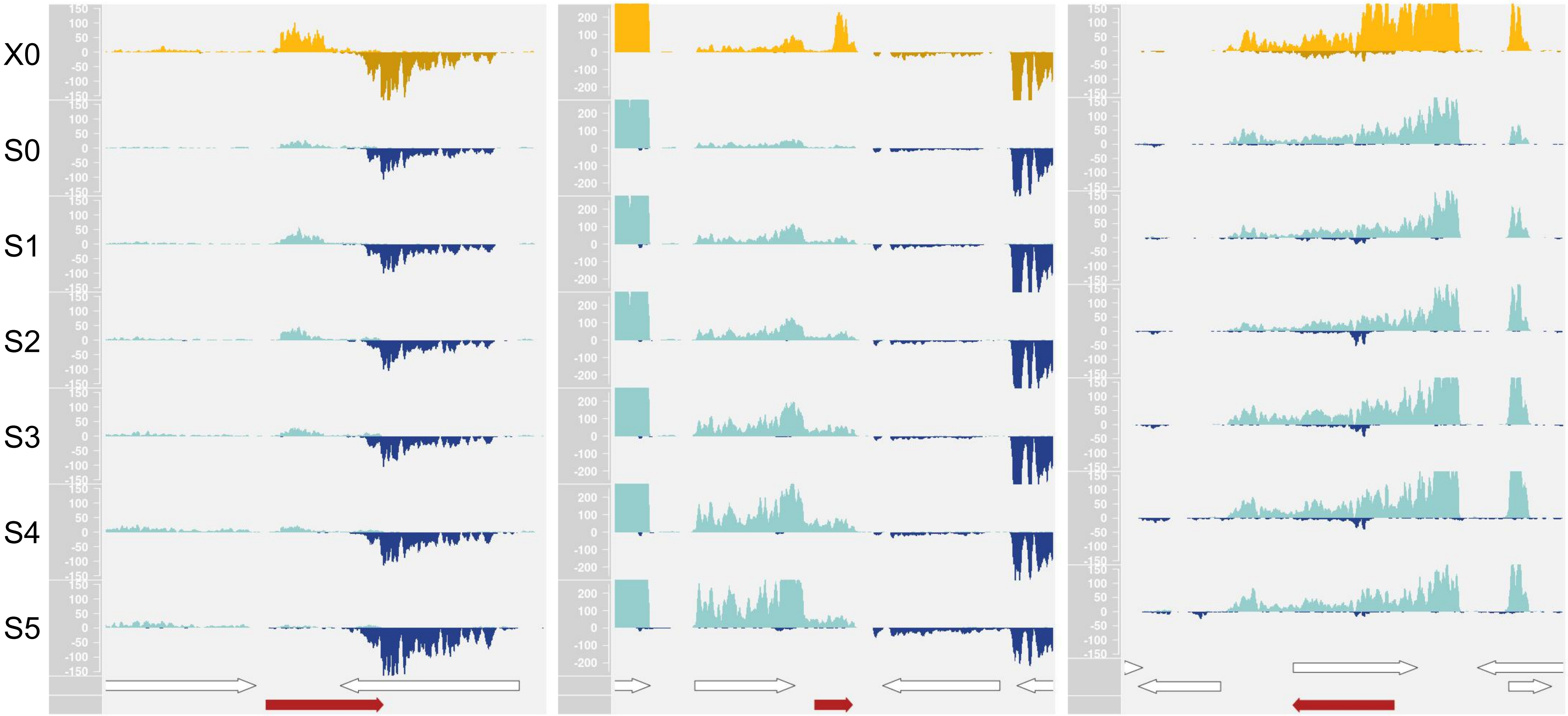

lncRNA-391 (XUT-0840)

lncRNA-413 (XUT-0905)

lncRNA-438 (XUT-0926)

Fig. S2. (continued)

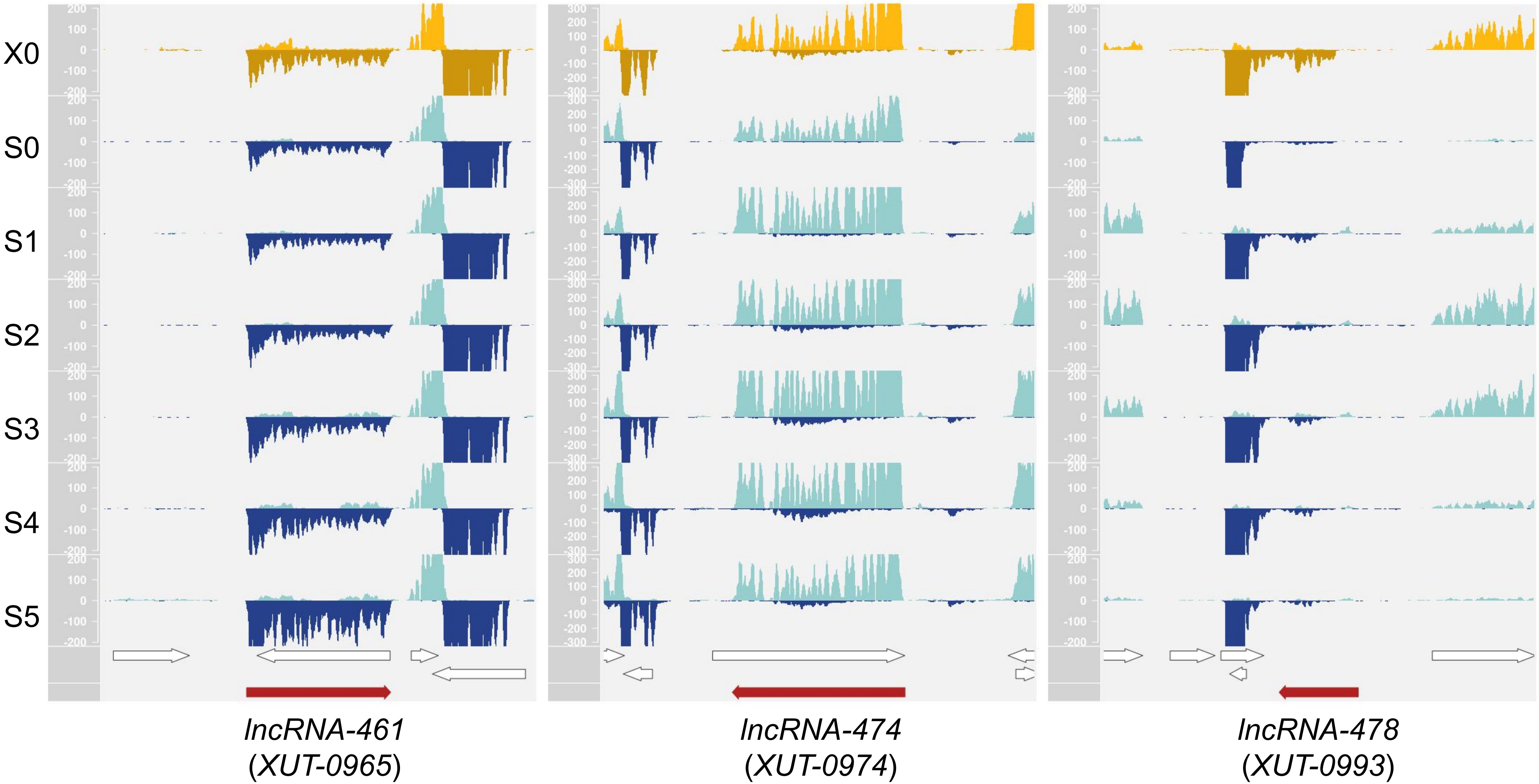

Fig. S2. (continued)

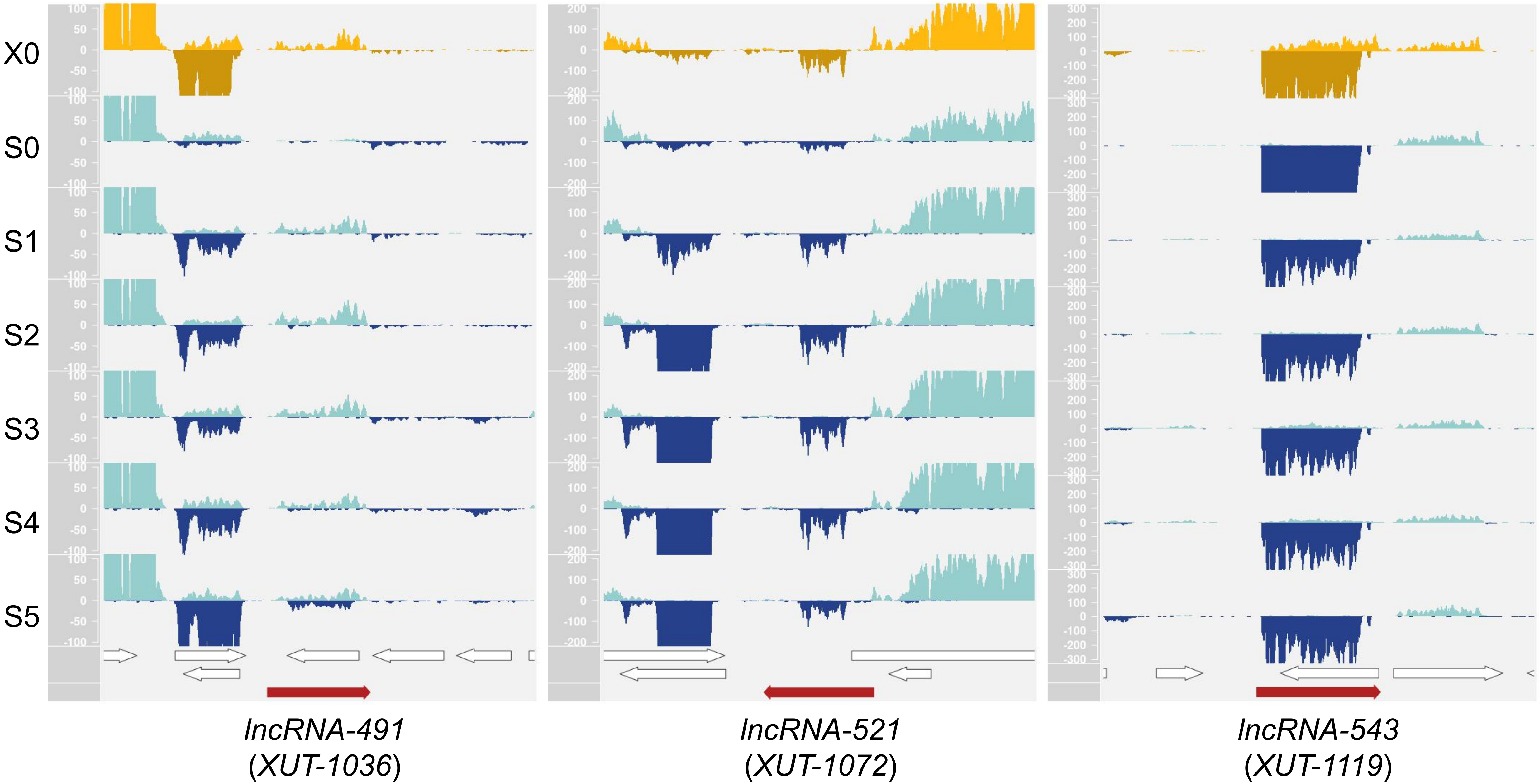

Fig. S2. (continued)

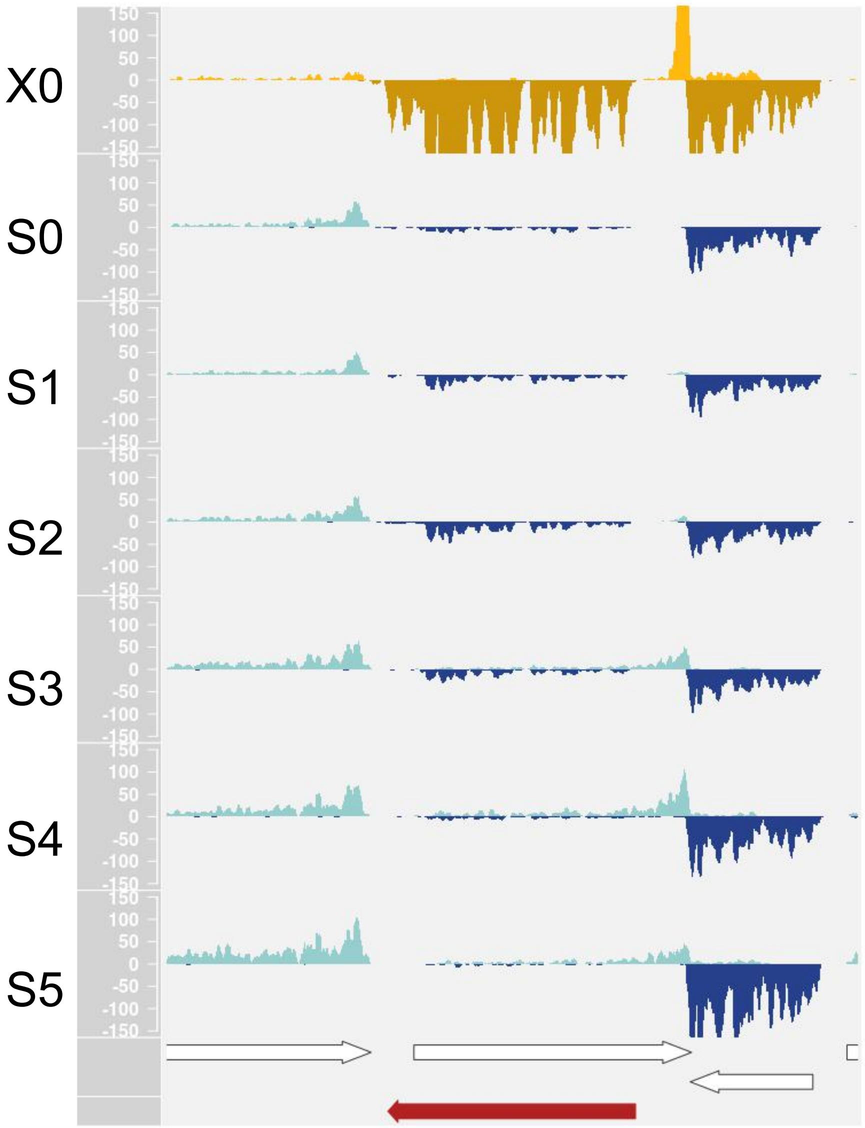

lncRNA-549
(*XUT-0055*)
