# Supplementary material for: Developmental Dynamics of Long Noncoding RNA Expression during Sexual Fruiting Body Formation in Fusarium graminearum
Source: mBio. 2018 Aug 14;9(4):e01292-18. doi: 10.1128/mBio.01292-18 (PMC6094484; doi:10.1128/mBio.01292-18)
Supplement: TABLE S3 [file mbo004184025st3.docx]

**Table S3.** Primers used in this study.

| **Primer** | **Sequence (5'-3')** | **Description** |
| --- | --- | --- |
| Qt | GAGCAGAGTCACGAGGACTCGACATCCGACTTTTTTTTTTTTTTTTTVN | for reverse transcription, modified  from Scotto-Lavino *et al*. 2006 |
| Qo | GAGCAGAGTCACGAGGACTCG | pair with GSP1 for 3'RACE |
| Qi | CACGAGGACTCGACATCCGAC | pair with GSP2 for 3'RACE |
| GSP1-cenp_T | GCTTGCTTTTGGGTGCTAGTGC | pair with Qo for 3'RACE |
| GSP2-cenp_T | GACCGAGGTGTCGCAGAACATC | pair with Qi for 3'RACE |
| GSP1-hir1 | CTGAGAAACTCGGGTGTCGGAAC | pair with Qo for 3'RACE |
| GSP2-hir1 | CGGAACAACACCGTGCTGTAC | pair with Qi for 3'RACE |
| GSP1-nse4 | CCAAACGCTCACCGAGTTCTTC | pair with Qo for 3'RACE |
| GSP2-nse4 | CAAAGCTGTAAAGCCCAGAGACC | pair with Qi for 3'RACE |
| GSP1-orc1 | CAAGCTCATCCATGAGCACTACGC | pair with Qo for 3'RACE |
| GSP2-orc1 | GCAGGGTATACGACGAGGACTAGG | pair with Qi for 3'RACE |
| GSP1-orc2 | GGCTGATCGTACACCAATATCCCAG | pair with Qo for 3'RACE |
| GSP2-orc2 | CCAGAGTAGCGCAAAGTCTGGC | pair with Qi for 3'RACE |
| GSP1-orc4 | GGCAGCGAAAGAGATCCATTCG | pair with Qo for 3'RACE |
| GSP2-orc4 | GTGGACAGCTCGTCGATAGATTCTTG | pair with Qi for 3'RACE |
| GSP1-rmd1 | CAAAGGCCAGGTGTGCAACC | pair with Qo for 3'RACE |
| GSP2-rmd1 | CTGACTGTTCCGTCTTCTTAGCTTG | pair with Qi for 3'RACE |
| GSP1-sor1 | GATCACCAGCCTTGAGGGTCTTG | pair with Qo for 3'RACE |
| GSP2-sor1 | CTTGACCTTGTCGCCAACCTCAAC | pair with Qi for 3'RACE |
| HYG5 | GCTTGGCTGGAGCTAGTGGAG | hph cassette amplification |
| HYG3 | CGGTCGGCATCTACTCTATTCCTT |  |
| YG-F | CGATGTAGGAGGGCGTGGATATGTC | split-marker construct pair with N3 |
| HY-R | TGTAGTGTATTGACCGATTCCTTGCG | split-marker construct pair with N5 |
| L5-dbp2 | GGAGGAGTGTCTTGGTTGATGAACG | left flanking region amplification |
| L3-dbp2 | GTTGACCTCCACTAGCTCCAGCCAAGCGAAGGATGAAGACGGAGCCGAATGG |  |
| R5-dbp2 | GGCAAAGGAATAGAGTAGATGCCGACCGGTGTGGTCTTCCATCATGAGGTCTTG | right flanking region amplification |
| R3-dbp2 | CGAAATATCTGTCACGATGTGCGTGG |  |
| N5-dbp2 | ACAATACGTTTCTGGATTGCTGGGAG | split-marker construct pair with HY-R |
| N3-dbp2 | CGCAGGAAGACACCACATAGCTGTC | split-marker construct pair with YG-F |
| GSPfwd-dbp2 | GTGACCGCAACGGTGGTGGTTTC | target gene amplification |
| GSPrev-dbp2 | CCTGAGCCTTGACCTCATCCATAAC |  |

| **Primer** | **Sequence (5'-3')** | **Description** |
| --- | --- | --- |
| L5-mtr4 | CCAAACAACAAACCCAGTTCGTGAG | left flanking region amplification |
| L3-mtr4 | GTTGACCTCCACTAGCTCCAGCCAAGCCATCCATATTGGCGATATTTTACCCGC |  |
| R5-mtr4 | GGCAAAGGAATAGAGTAGATGCCGACCGCCTTTTGCTCGTAGAATGGACTGCC | right flanking region amplification |
| R3-mtr4 | CCTCAGGATGTGAATGGAGATGACTC |  |
| N5-mtr4 | GAGAAACCACGCACCAGCAATG | split-marker construct pair with HY-R |
| N3-mtr4 | CACCTTCCGAAATTGCGACGC | split-marker construct pair with YG-F |
| GSPfwd-mtr4 | GACCGATTCTTCAACGAGCAAACC | target gene amplification |
| GSPrev-mtr4 | GCAACGATATCTCTGCGGATCTTG |  |
| L5-rrp6 | GTCTCCACACGAGTGATTCAAAGC | left flanking region amplification |
| L3-rrp6 | GTTGACCTCCACTAGCTCCAGCCAAGCGTTGCGACAAACAAGTGCGCGTTG |  |
| R5-rrp6 | GGCAAAGGAATAGAGTAGATGCCGACCGCCGGGTAATCATGGCAAAACTAGGAG | right flanking region amplification |
| R3-rrp6 | GACTTTCTAGGCTGCGGTTGCC |  |
| N5-rrp6 | GGTTGGCAGACAGGTACTTTACC | split-marker construct pair with HY-R |
| N3-rrp6 | CTACGCCTGTTGAACTCAGAGACC | split-marker construct pair with YG-F |
| GSPfwd-rrp6 | CATCCGAAACGCCAACATCTCC | target gene amplification |
| GSPrev-rrp6 | CTTGGTTGAGAACCTCGAGTTTGTG |  |
| L5-xrn1 | GCACGGCTGAGAATATAGCAAGTCTC | left flanking region amplification |
| L3-xrn1 | GTTGACCTCCACTAGCTCCAGCCAAGCGACAGTGGACAATTTCCAGTCCAC |  |
| R5-xrn1 | GGCAAAGGAATAGAGTAGATGCCGACCGCTGAAGGGCGGTCTCCTGTGATATC | right flanking region amplification |
| R3-xrn1 | CACTACTACAGCACCAAGAAGGAGG |  |
| N5-xrn1 | CGTTGATGTGTAGAAGGCTGAAAGGC | split-marker construct pair with HY-R |
| N3-xrn1 | GACGCCGATCTGAGAAGCCTCTTC | split-marker construct pair with YG-F |
| GSPfwd-xrn1 | TGCGGCTTACCGAGTAATGAAGAC | target gene amplification |
| GSPrev-xrn1 | GTGCCTTTTCATCGGGTTCCAAC |  |
